# A Noble AuPtAg‐GOx Nanozyme for Synergistic Tumor Immunotherapy Induced by Starvation Therapy‐Augmented Mild Photothermal Therapy

**DOI:** 10.1002/advs.202202332

**Published:** 2022-09-25

**Authors:** Man Wang, Mengyu Chang, Pan Zheng, Qianqian Sun, Guangqiang Wang, Jun Lin, Chunxia Li

**Affiliations:** ^1^ Institute of Molecular Sciences and Engineering Institute of Frontier and Interdisciplinary Science Shandong University Qingdao 266237 P. R. China; ^2^ State Key Laboratory of Rare Earth Resource Utilization Changchun Institute of Applied Chemistry Chinese Academy of Sciences Changchun 130022 P. R. China; ^3^ Department of Respiratory Medicine Qilu Hospital Shandong University Qingdao 266071 P. R. China

**Keywords:** cascade reactor, immune system activation, mild photothermal therapy, nanozymes, starvation therapy, synergistic therapy

## Abstract

Notwithstanding immune checkpoint blocking (ICB) therapy has made eminent clinical breakthroughs, overcoming immunologically “cold” tumors remains challenging. Here, a cascade potentiated nanomodulator AuPtAg‐GOx is engineered for boosting immune responsiveness. Upon 1064 nm laser irradiation, AuPtAg‐mediated mild photothermal therapy (PTT) activates cytotoxic T lymphocytes and reverses the immunogenic “cold” tumor microenvironment. Further, to amplify the thermal sensitivity of tumor cells, glucose oxidase (GOx) is introduced to suppress the production of heat shock proteins, thereby promoting mild photothermal therapy. Complementarily, AuPtAg nanozymes with catalase‐like activity can ameliorate tumor hypoxia, significantly improving the GOx activity. As a result, the combination of AuPtAg‐GOx with self‐augmented photothermal ability and PD‐L1 antibody can further escalate the antitumor efficacy. The AuPtAg‐GOx‐based synergistic starvation therapy, mild PTT, and immunotherapy cascade enhancement therapy strategy can be a favorable tool to effectively kill cancer cells.

## Graphical abstract

A cascade promoted AuPtAg‐glucose oxidase (GOx) nanomodulator is designed for synergistic starvation/photothermal/immunotherapy. The GOx‐mediated starvation therapy inhibits heat shock protein production for enhanced AuPtAg mediated mild photothermal therapy, which would sequentially activate the systemic immune response, prompting the tumor microenvironment to become “hot.” Therefore, AuPtAg‐GOx combined with ɑ‐PD‐1 ligand 1 could obviously inhibit tumor growth.

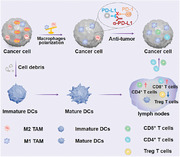

## 1 Introduction

Immunotherapy has emerged as an excellent cancer treatment strategy, which stimulates and maintains the systemic immune response through the host's own immune system to resist the spread of tumor cells.^[^ ^1^ ^]^ Among various immunotherapies, immune checkpoint blockade (ICB) therapy has gained important clinical advances and revolutionary breakthrough.^[^ ^2^ ^]^ Since 2011, FDA‐approved ipilimumab (anti‐CTLA4) has been used for the treatment of metastatic melanoma,^[^ ^3^ ^]^ monoclonal antibodies targeting programmed cell death 1 (PD‐1) and PD‐1 ligand 1 (PD‐L1) axis,^[^ ^4^ ^]^ indoleamine 2,3‐dioxygenase inhibitors,^[^ ^5^ ^]^ CD47 antibodies,^[^ ^6^ ^]^ etc. were successively developed for cancer immunotherapy.^[^ ^7^ ^]^ Among them, anti‐PD‐L1/anti‐PD‐1 antibodies have become the most widely used immune checkpoint inhibitors.^[^ ^8^ ^]^ However, the efficacy of monomodal ICB therapy is frequently modest due to immunologically “cold” tumors and low tumor‐infiltrating lymphocytes (TILs).^[^ ^9^ ^]^ Fortunately, the inadequacy of ICB‐based immunotherapy can be remedied by synergistic therapy, which has brought an incredible revolution in cancer therapy.^[^ ^10^ ^]^ For example, Yan et al. designed an aggregation‐induced emission reagent (TPA‐BT‐DPTQ)‐mediated PTT to eliminate primary tumors and boost immunogenicity. Synergistic PTT and PD‐L1 antibody successfully avoided metastasis and recurrence of cancer.^[^ ^11^ ^]^ Additionally, Liang et al. designed a nanoaircraft carrier to deliver miscellaneous therapeutic agents, achieving modulation of the immune response to facilitate ICB therapy.^[^ ^12^ ^]^ Therefore, it is a promising strategy to design a synergistic therapy that can reshape the tumor microenvironment (TME) and improve the TILs level in solid tumors.

Photothermal therapy (PTT), a spatiotemporally controllable noninvasive modality, has attracted increasing attention.^[^ ^13^ ^]^ During PTT, photothermal conversion agents can transform light into hyperthermia to ablate the tumor cells.^[^ ^14^ ^]^ Importantly, hyperthermia can improve the immunogenicity of the tumor and create a favorable niche for TILs recruitment, thereby facilitating ICB therapy.^[^ ^15^ ^]^ However, to realize ideal tumor damaging effect, the hyperthermia over 50 °C is essential, which inevitably injures the healthy tissues around the tumors.^[^ ^16^ ^]^ As an alternative, mild PTT (40–43 °C) is more preferable for clinical applications. Nevertheless, the up‐regulated protective heat shock proteins (HSPs) within cancer cells and PD‐L1 expressed on the surface of cancer cells induced by heat stress often cause suboptimal PTT effect.^[^ ^17^ ^]^ To shrinking the thermoresistance of cancer cell, a number of small molecule HSPs inhibitors (GA, 17‐AAG etc.) have been exploited.^[^ ^18^ ^]^ Regrettably, these HSPs inhibitors can only inhibit the activity of the already generated HSPs after heat stimulus rather than before therapy,^[^ ^19^ ^]^ resulting in delayed therapeutic effect. Furthermore, these small molecular inhibitors lack generalizability, because one inhibitor can only target one specific HSP. Therefore, inhibiting all kinds of HSPs production at the source is more beneficial to improve the effect of mild PTT.

Glucose oxidase (GOx), a prominent endogenous oxidoreductase for cancer starvation therapy, can deplete intracellular glucose and O_2_ to produce H_2_O_2_ and gluconic acid.^[^ ^20^ ^]^ By consuming glucose, the GOx can effectively inhibit the production of intracellular adenosine triphosphate (ATP).^[^ ^21^ ^]^ As we all know, the expression of HSP was highly positively correlated with ATP content.^[^ ^22^ ^]^ Therefore, inhibiting the production of intratumoral ATP by GOx‐mediated starvation therapy can be a promising method to overcome HSPs‐induced tumor thermoresistance. However, the hypoxic TME severely constrains the activity of GOx. As a consequence, ameliorating hypoxia is crucial to optimize the effects of GOx. To solve the encountered limitations, noble metal nanozymes with extraordinary catalase (CAT)‐like activity and impressive photothermal conversion efficiency is an ideal candidate.^[^ ^23^ ^]^ Therefore, integrating GOx with noble metal nanozymes is an intelligent strategy for synergistically starvation/mild photothermal/immunotherapy.

Based on the above considerations, a noble AuPtAg‐GOx nanozyme was constructed for synergistic tumor immunotherapy induced by starvation therapy‐augmented mild photothermal therapy. The AuPtAg was first prepared by a simple one pot method and then GOx was covalently connected to its surface with SH‐PEG‐NH_2_ as a bridge. After the nanoparticles were endocytosed into tumor cells, i) AuPtAg‐GOx with CAT‐like activity can catalyze intratumoral overexpressed H_2_O_2_ into O_2_. Then, AuPtAg‐GOx can realize a remarkable starvation therapy effect by consuming the glucose under the assistance of O_2_. What is more, the cutoff of nutrients cause a reduced intratumoral ATP level. ii) Upon 1064 nm laser irradiation, mild heat is generated by AuPtAg‐GOx in the tumor area. Meanwhile, the limited ATP level can inhibit the synthesis of HSPs, thus realizing the significant therapeutic effect of mild PTT. iii) Mild‐PTT can intensify the recruitment of TILs to reprogram the “cold” TME, sensitizing the tumor to ICB therapy. Therefore, by integrating AuPtAg‐GOx with ɑ‐PD‐L1, both primary and distant tumors can be effectively inhibited. In a nutshell, AuPtAg‐GOx can achieve mild PTT augmented by starvation therapy, and further promotes immunotherapy effects through mild PTT. Such cascade promoting synergistic strategies may be generally applicable for high‐efficient cancer therapy.

## 2 Results and Discussion

**Scheme 1:**
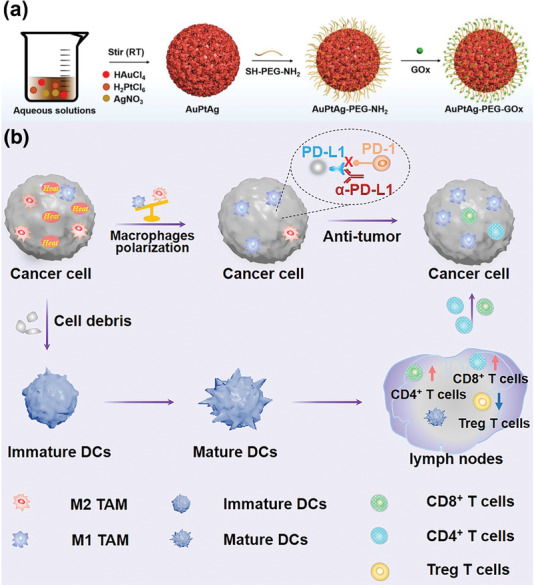
a) Schematic illustration of the formation of AuPtAg‐GOx nanozyme and b) the mechanism for tumor immunotherapy induced by starvation therapy‐augmented mild photothermal therapy.

**Figure 1:**
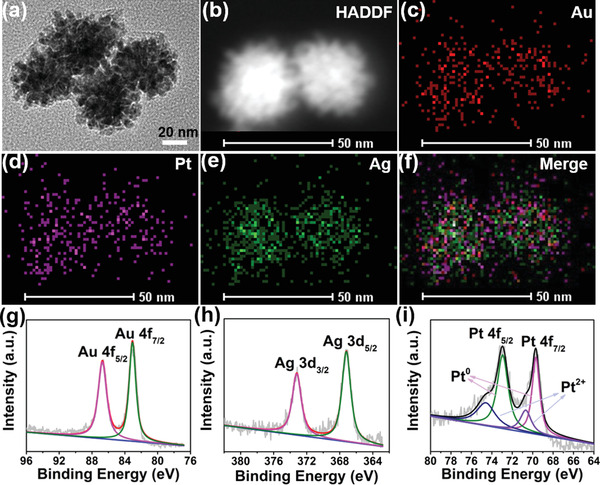
The characterization of AuPtAg. a) TEM image, b–f) the high‐angle annular dark‐field scanning transmission electron microscopy (HAADF‐STEM) and elemental mapping images, high‐resolution XPS spectra of g) Au 4f, h) Ag 3d, and i) Pt 4f.

The AuPtAg nanozymes were synthesized by a one‐step method,^[^ ^24^ ^]^ and the major experimental procedures were shown in **Scheme** **1**. In detail, L‐proline was employed as a chelator for modulating the topography due to its selective adsorption onto the high index plane of the metal surface. Subsequently, the metal precursors (PtCl_6_^2−^, Ag^+^ and AuCl_4_^−^) were gradually reduced by L‐ascorbic acid. A series of AuPtAg with different molar ratios of Au, Pt, and Ag were synthesized, and the optimal ratio of AuPtAg (Au:Pt:Ag = 1:1:1.22) was selected for subsequent tests (Table S1 and Figures S1–S3, Supporting Information). Dendritic AuPtAg nanozymes with a mean diameter of 45 nm was monitored by transmission electron microscopy (TEM, **Figure** **1**a). High‐angle annular dark‐field scanning transmission electron microscopy (HAADF‐STEM) images showed that Au, Pt, and Ag atoms were homogeneously dispersed in the nanozymes (Figure 1b–f). High‐resolution TEM (HRTEM) images indicated that the lattice fringes spacing was 0.212, 0.221, and 0.212 nm, corresponding to the (111) plane of Au, Pt, and Ag, respectively (Figure S4, Supporting Information). Consistent with the HRTEM results, the X‐ray diffraction (XRD) patterns indicated that the main peaks of AuPtAg were 38.38°, 44.8°, 64.7°, 78.12°, and these peaks appeared between bulk Au, Pt and Ag, showing their alloying nature (Figure S5, Supporting Information). The X‐ray photoelectron spectroscopy (XPS) was performed to determine the elemental valence states (Au, Pt, and Ag elements) of AuPtAg. As shown in Figure 1g–i, the Au^0^, Ag^0^ and Pt^0^ coexisted in AuPtAg, which further verified the alloy properties of AuPtAg. To enhance the biological compatibility of AuPtAg, SH‐PEG‐NH_2_ was linked on its surface. After SH‐PEG‐NH_2_ was modified, the zeta potential decreased from ‐0.82 to ‐10.93 mV (Figure S6, Supporting Information), the change in zeta potential demonstrated the successful modification of SH‐PEG‐NH_2_. GOx, an efficient natural enzyme for starvation therapy, was subsequently covalently attached on AuPtAg‐PEG‐NH_2_. The TEM image of AuPtAg‐GOx is shown in Figure S7 (Supporting Information) and there was no obvious morphology change after modification of PEG and GOx. The zeta potential and mean hydrodynamic size of the proposed AuPtAg‐GOx were ‐12 mV and 164 nm (Figure S8, Supporting Information), respectively. Based on the Bradford protein assay, 10 mg of AuPtAg‐PEG‐NH_2_ was attached with 0.61 mg of GOx. The AuPtAg‐GOx showed good stability in water, PBS, and Roswell Park Memorial Institute (RPMI) culture medium, providing a possibility for further in vivo applications (Figure S9, Supporting Information).

**Figure 2:**
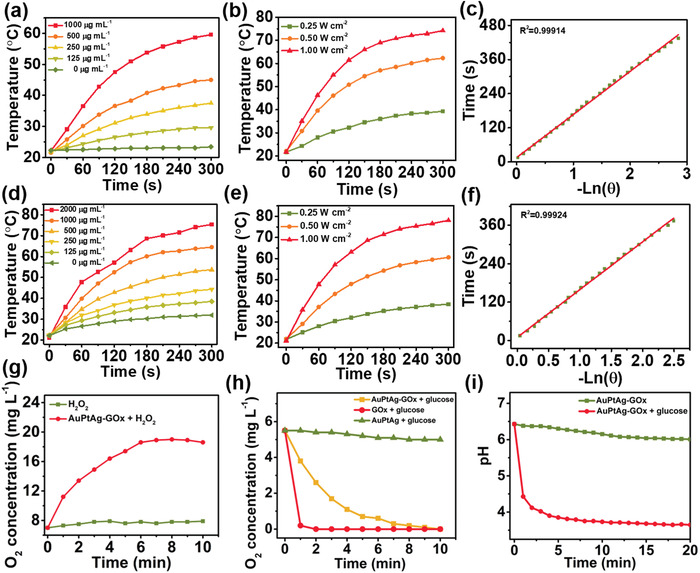
Temperature changes of AuPtAg at different concentrations under a) 808 nm and d) 1064 nm laser irradiation (0.5 W cm^−2^, 5 min). Temperature changes of AuPtAg (1000 µg mL^−1^) with various laser power density (808 nm laser (b) and 1064 nm laser (c) of for 5 min). Plot of cooling time versus negative natural logarithm of the temperature driving force (808 nm laser (c) and 1064 nm laser (f)). g) O_2_ generation curve of H_2_O_2_ solution (3.2 × 10^−3^ m) without and with addition AuPtAg‐GOx (200 µg mL^−1^). h) The oxygen concentration and i) pH plotted against time in different solutions (AuPtAg‐GOx: 250 µg mL^−1^, glucose: 3.88 × 10^−3^ m).

As shown in Figure S10 (Supporting Information), AuPtAg has a broad absorption band in the near‐infrared (NIR) region, and the absorption coefficients at 1064 nm and 808 nm are 2.71 L g^−1^ cm^−1^ and 4.01 L g^−1^ cm^−1^, respectively (Figure S11, Supporting Information), implying that AuPtAg has great potential as a photothermal agent. Subsequently, the photothermal conversion ability of AuPtAg at 808 nm and 1064 nm was measured by infrared thermal camera. When different concentrations of AuPtAg were exposed to 808 nm or 1064 nm lasers (0.5 W cm^−2^) for 5 min, a significant concentration‐dependent temperature rise was observed (**Figure** **2**a,b). Corresponding infrared images are shown in Figures S12 and S13 (Supporting Information). The change of pure water was not obvious, indicating that AuPtAg has the ability to quickly and efficiently convert 808 and 1064 nm NIR light energy into heat energy. Then, the NIR photothermal capability of AuPtAg was further detected in detail under 808 nm or 1064 nm continuous wave laser irradiation at various laser power densities (0.25, 0.5, and 1.0 W cm^−2^), which also indicated the strong laser‐power‐dependent photothermal effect of AuPtAg (Figure 2d,e), and the maximum temperature rise is as high as 64.5 °C (1064 nm, 1 W cm^−2^). The photothermal conversion efficiencies of AuPtAg at 808 nm and 1064 nm were calculated to be 47% and 41% (Figure 2c,f), respectively (according to previously reported methods,^[^ ^25^ ^]^ detailed calculation process in supporting information). In the case of little difference in photothermal conversion efficiency at 808 nm and 1064 nm, NIR‐II light (1064 nm) with inherent advantages, such as deeper tissue penetration and higher maximum permissible exposure (1 W cm^−2^), was selected for cancer treatment (American National Standard for Safe Use of Lasers, ANSI Z136.1‐2007).^[^ ^26^ ^]^ In addition, the photothermal properties of AuPtAg did not change significantly in the process of four laser switches, indicating that it has good photothermal stability (Figure S14, Supporting Information). All the above results indicated that AuPtAg was a promising candidate for PTT in NIR‐II bio‐window.

The CAT‐like activity of AuPtAg‐GOx was first verified, which can effectively convert H_2_O_2_ to O_2_. The concentration of dissolved oxygen increased gradually over 10 min in the AuPtAg‐GOx + H_2_O_2_ group, whereas it did not change significantly in the PBS + H_2_O_2_ group, suggesting that AuPtAg‐GOx had CAT‐like activity (Figure 2g). Then, the ability of AuPtAg‐GOx mediated glucose decomposition was verified. As shown in Figure S15 (Supporting Information), the glucose concentration in the solution was gradually decreasing as the reaction progressed (Figure S16, Supporting Information), indicating that AuPtAg‐GOx can effectively consume glucose to achieve starvation treatment. In addition, to further verify the occurrence of this reaction, we examined the changes of oxygen content and pH value in the solution. As displayed in Figure 2h, the oxygen concentration did not change obviously in the AuPtAg + glucose group. While in the GOx + glucose group, O_2_ content decreased rapidly. Moreover, the concentration of dissolved oxygen reduced slowly in the AuPtAg‐GOx + glucose group, which is because H_2_O_2_ generated during glucose oxidation (Figure S17, Supporting Information) would be reconverted to O_2_ by AuPtAg. What is more, the sharp decrease of pH in the AuPtAg‐GOx + glucose group also proved the production of gluconic acid (Figure 2i). Because the CAT‐like activity of AuPtAg and activity of GOx can be effectively maintained in a certain glucose concentration range (Figure S18, Supporting Information) and 37–42 °C range (Figure S19, Supporting Information), AuPtAg‐GOx has great potential for synergistic mild PTT/starvation treatment.

**Figure 3:**
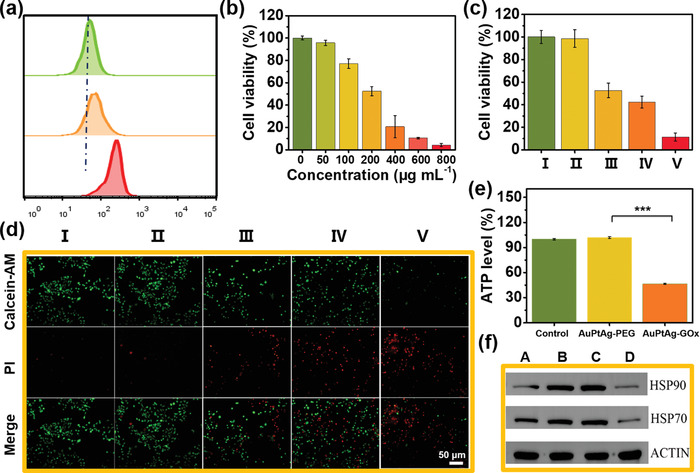
a) Flow cytometric assay of 4T1 cells incubated with AuPtAg‐GOx‐RhB for different time periods. b) Viability of 4T1 cells treated with different concentrations of AuPtAg‐GOx. c) Cell viabilities and d) corresponding fluorescence images of 4T1 cells constrained with calcein‐AM (live cells, green) and propidium iodide (PI, dead cells, red) after being treated with different conditions. Group I: Control; II: 1064 nm (0.5 W cm^−2^); III: AuPtAg‐GOx; IV: AuPtAg‐PEG + 1064 nm (0.5 W cm^−2^); V: AuPtAg‐GOx + 1064 nm (0.5 W cm^−2^). e) Intracellular ATP level after different treatments. f) Western blots of HSP70 and HSP90 after different treatments. Group A: Control; B: 42 °C incubation; C: AuPtAg‐PEG + 1064 nm (0.5 W cm^−2^); D: AuPtAg‐GOx + 1064 nm (0.5 W cm^−2^). Statistical significance is assessed by an unpaired Student's two‐sided *t*‐test. ***p < 0.001.

The cellular uptake behavior of AuPtAg‐GOx was first researched before cancer cell treatment with AuPtAg‐GOx. Rhodamine B (RhB) was attached on AuPtAg‐GOx for the labeling of AuPtAg‐GOx, as shown in the **Figure** **3**a, the internalization amount of AuPtAg‐GOx increased with time. And the picture of the fluorescence microscope after 6 h internalization was shown in Figure S20 (Supporting Information). Then, the biocompatibility of AuPtAg‐PEG was tested, after 4T1 cells and L929 cells were incubated with AuPtAg‐PEG (500 µg mL^−1^), the cell viabilities were still as high as 85%, indicating that AuPtAg‐PEG has low cytotoxicity (Figure S21, Supporting Information). Whereafter, the anticancer effect of AuPtAg‐GOx in vitro was evaluated on 4T1 cells. In the absence of laser irradiation, the toxicity to 4T1 cells is mainly dependent on the dosage of AuPtAg‐GOx, with a half maximal inhibitory concentration (IC50) around 199.34 µg mL^−1^ (Figure 3b, Figure S22, Supporting Information). As shown in Figure 3c, the 4T1 cells treated with only 1064 nm laser irradiation (0.5 W cm^−2^) achieved high viability (>98%), indicating that light alone cannot kill cells. While 47% of 4T1 cells were killed in the AuPtAg‐GOx (200 µg mL^−1^) group. Notably, 89% cancer cells growth inhibition was achieved in the AuPtAg‐GOx (200 µg mL^−1^) + 1064 nm group (Figure 3c). This is because starvation therapy not only starves tumor cells, but also reduces the production of HSPs, tremendously promoting the effect of mild PTT. The similar results were further demonstrated by the live‐dead cell staining assays (Figure 3d). Then, the promoting mechanism of starvation therapy on mild‐PTT was investigated. To verify that AuPtAg‐GOx would shorten the lever of ATP in 4T1 cells, the intracellular ATP levels were measured after different treatments. As shown in Figure 3e, the level of ATP did not change significantly in the AuPtAg‐PEG group. While in the AuPtAg‐GOx group, the ATP content declined obviously, indicating that AuPtAg‐GOx could reduce intracellular ATP content with high efficiency by glucose consumption. Subsequently, the levels of the HSP90 and HSP70 in 4T1 cells with different treatments were further examined by Western blot. As expected, mild‐PTT (42°C incubation group and AuPtAg‐PEG + 1064 nm group) would elevate the expression of HSP, while the expression of HSP70 and HSP90 was significantly lessened in AuPtAg‐GOx + 1064 nm group (Figure 3f). The above results indicated that starvation therapy could effectively inhibit the rise of HSP induced by mild‐PTT, so as to reduce the thermoresistance of 4T1 cells. Taken together, the effect of mild‐PTT was greatly enhanced by the synergistic effect of starvation therapy.

**Figure 4:**
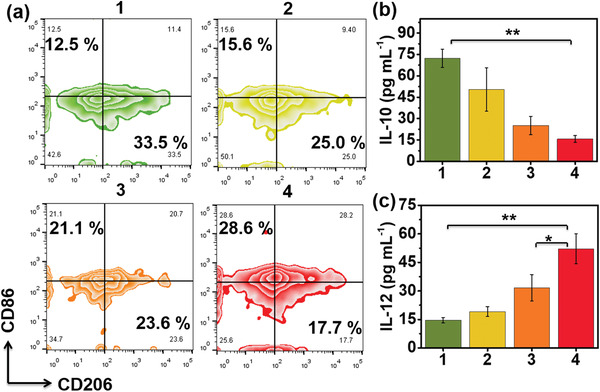
Immune stimulation effect of AuPtAg‐GOx in vitro. a) Representative flow cytometer plots of M2‐type macrophages (CD206^+^) and M1‐type macrophages (CD86^+^) in TAMs (F4/80^+^). Secretion levels of b) IL‐10 and c) IL‐12 in the supernatant after different treatments. Groups: 1) control, 2) AuPtAg‐GOx, 3) AuPtAg‐PEG + 1064 nm (0.5 W cm^−2^), 4) AuPtAg‐GOx + 1064 nm (0.5 W cm^−2^). Statistical significance is assessed by an unpaired Student's two‐sided *t*‐test. *p < 0.05, **p < 0.01.

It has been reported that photothermal can effectively reverse the immunosuppressive TME.^[^ ^27^ ^]^ Subsequently, the ability of AuPtAg‐GOx to reprogram “cold” tumors into “hot” tumors was researched. Tumors are “cold” in immunology, which largely depends on the infiltration of immunosuppressive M2 phenotype tumor‐associated macrophages (TAMs).^[^ ^28^ ^]^ Hence, whether AuPtAg‐GOx could reprogram the macrophage polarization from M2 to M1 was measured in vitro (Figure S23, Supporting Information). M2 macrophages were first procured by pretreatment of RAW264.7 macrophages with interleukin‐4 (IL‐4). In AuPtAg‐PEG + 1064 nm group, the proportion of M2 macrophages decreased from 33.5% to 23.6%, while M1 macrophages increased from 12.5% to 21.1%. In AuPtAg‐GOx + 1064 group, M2 macrophages declined by 5.9% (from 23.6% to 17.7%) and M1 macrophages ascended by 7.5% (from 21.1% to 28.6%) compared with the AuPtAg‐PEG + 1064 group (**Figure** **4**a). Similarly, the secretion of IL‐10 by M2 macrophage in the AuPtAg‐GOx + 1064 nm group was 0.21‐fold of that in the control group (Figure 4b), while the secretion of IL‐12 by M1 macrophages was 3.6‐fold (Figure 4c). The content of IL‐10 in the AuPtAg‐GOx + 1064 nm group was 0.35‐fold of that in the control group and that of IL‐12 was 2.2‐fold, all above results demonstrating that starvation therapy facilitated the photothermal effect of AuPtAg, which could polarize M2 macrophages to M1 macrophages. It has been reported that the immunogenic cell death (ICD) is an important trigger for anti‐tumor immune responses in vivo.^[^ ^29^ ^]^ The ICD induced by AuPtAg‐GOx was examined through measuring the release of three markers, ATP, calreticulin (CRT), and high mobility group box 1 (HMGB‐1). Compared with the control group, the AuPtAg‐GOx + 1064 nm group showed significant ATP release (Figure S24, Supporting Information), HMGB‐1 release (Figure S25, Supporting Information), and CRT exposure (Figure S26, Supporting Information), indicating that AuPtAg‐GOx can effectively promote ICD under laser irradiation.

**Figure 5:**
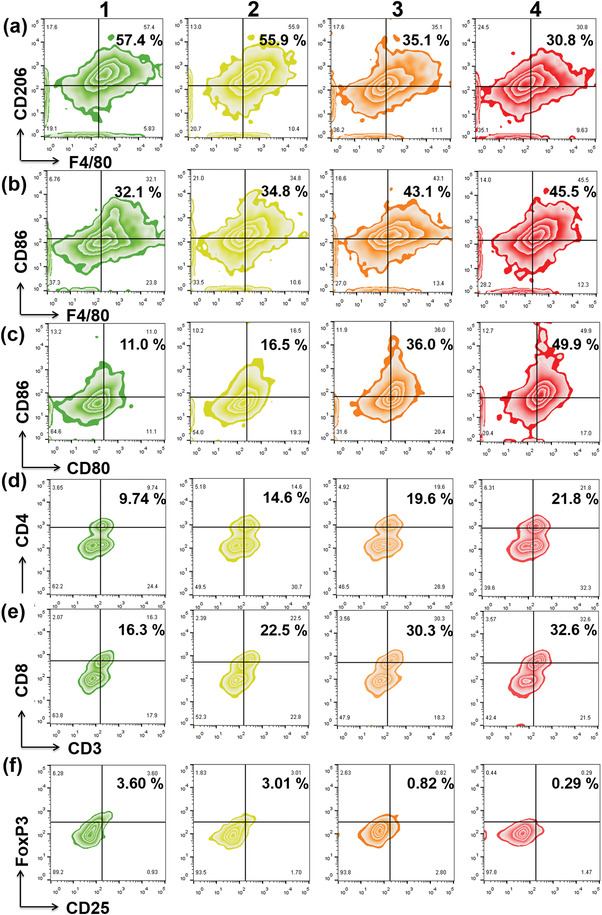
Flow cytometric analyses of the populations of a) M2 and b) M1 macrophages in tumor. c) The populations of DC cells in lymph nodes. The populations of d) CD4^+^ T cells, e) CD8^+^ T cells, and f) Treg in splenocytes after different treatments. Groups: 1) control, 2) AuPtAg‐GOx, 3) AuPtAg‐PEG + 1064 nm (0.7 W cm^−2^), 4) AuPtAg‐GOx + 1064 nm (0.7 W cm^−2^).

Encouraged by the excellent ability to reverse immunosuppressive TME in vitro, the capacity of AuPtAg‐GOx to strengthen immunoreaction was further measured in 4T1 tumor‐bearing Balb/C mice. As expected, both AuPtAg + 1064 nm group and AuPtAg‐GOx + 1064 nm group could remarkably downregulate the proportion of M2 macrophages and increase the proportion of M1 macrophages in tumors. Furthermore, the effect was more obvious in the group of AuPtAg‐GOx + 1064 nm due to the synergistic effect of starvation therapy, the ratio of M2 macrophages shortened from 57.4% to 30.8% (**Figure** **5**a), M1 macrophages risen from 32.1% to 45.5% (Figure 5b). All the above results testified that combination therapy could effectively switch M2 phenotype TAMs to M1 macrophages. To further explore the competence of combination therapy to reinforce tumor immune responses, the percentage of dendritic cells (DC cells, an antigen‐presenting cell) maturation was tested under the stimulation of various synergistic therapies. As shown in Figure 5c, the proportion of DC maturation in tumor‐infiltrating lymph nodes increased by 25% (from 11% to 36%) in the AuPtAg + 1064 nm treatment group. In the AuPtAg‐GOx + 1064 nm group, the DC cell maturation rate was found to be more signally grown (from 11% to 49.9%). Since DCs maturation would boost T cell activation, the T cell activation situation was tested by flow cytometry in the spleen, the largest immune organ in the body. In line with DC maturation analysis, compared with control group, CD4^+^ T cells increased by 9.86% and 12.06% (Figure 5d), and CD8^+^ T cells elevated by 14% and 16.3% (Figure 5e, Figure S27, Supporting Information) in AuPtAg‐PEG + 1064 nm treatment and AuPtAg‐GOx + 1064 nm treatment groups, respectively. In addition, the proportion of regulatory T cells (Treg T cells, an immune cell that negatively regulates immune responses) was analyzed in the spleen by flow cytometry. The number of Treg T cells was efficiently suppressed in both the AuPtAg + 1064 nm treatment group and the AuPtAg‐GOx + 1064 nm treatment group in Figure 5f and Figure S28 (Supporting Information). Moreover, the levels of IL‐6 and TNF‐*α* were examined by ELISA, and both AuPtAg + 1064 nm and AuPtAg‐GOx + 1064 nm treatment groups promoted IL‐6 and TNF‐*α* secretion compared to the control group (Figure S29, Supporting Information). However, the cytokine levels stimulated by AuPtAg‐GOx + 1064 nm were obviously higher than those by AuPtAg + 1064, suggesting that AuPtAg‐GOx + 1064 nm could trigger a stronger immune response. All above results demonstrated that AuPtAg‐GOx based starvation therapy‐augmented mild PTT could re‐establish the immunosuppressive TME to elicit a robust antitumor immune response, offering great promise for eliminating primary and metastatic tumors.

**Figure 6:**
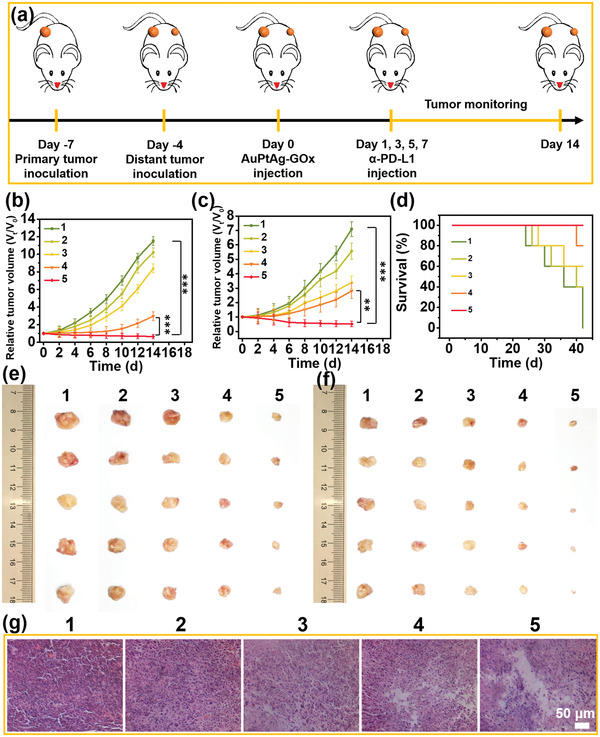
a) Scheme of synergistic treatment combined with *α*‐PD‐L1 blockade treatment for bilateral tumor models. b) The tumor sizes of primary tumor volume and c) distant tumor volume in Balb/c mice with different treatments. d) Survival rate of Balb/c mice after various treatments. Photographs of dissected e) primary tumors and f) distal tumors from mice at the end of intravenous injection treatment (day 14). g) H&E staining images of the dissected tumor tissues after 14 d of treatment. Data are presented as mean ± s.d. (*n* = 5). Groups: 1) control, 2) AuPtAg‐GOx, 3) AuPtAg‐PEG + 1064 nm (0.7 W cm^−2^), 4) AuPtAg‐GOx + 1064 nm (0.7 W cm^−2^), 5) AuPtAg‐GOx + 1064 nm + ɑ‐PD‐L1 (0.7 W cm^−2^). Statistical significance is assessed by an unpaired Student's two‐sided *t*‐test. **p < 0.01. ***p < 0.001.

Inspired by the excellent ability to ablate 4T1 cells and immune activation property of AuPtAg‐GOx, the in vivo antitumor performance of AuPtAg‐GOx was evaluated in bilateral tumor‐bearing mice (**Figure** **6**a). To determine that AuPtAg‐GOx can accumulate in tumors, the distribution of AuPtAg‐GOx in different organs and tumors was measured. After injection of AuPtAg‐GOx, the content of Au in tissues at different time points was detected by inductively coupled plasma mass spectrometry (ICP‐MS). Ideally, AuPtAg‐GOx could aggregate in the tumor through enhanced permeability and retention (EPR) effect, and the content is the highest in the tumor 12 h after injection (Figure S30, Supporting Information). It was further verified by photothermal imaging, when the mice injected with AuPtAg‐GOx and saline for 12 h were exposed to 1064 nm laser irradiation, the temperature of the tumor part of the mice increased gradually in the AuPtAg‐GOx group, while there was no obvious change in the saline group (Figure S31, Supporting Information). The above results proved that AuPtAg‐GOx could aggregate in the tumor and could generate mild heat (43 °C) by adjusting the laser power density. For better inhibition of tumor growth and metastasis, immune checkpoint inhibitors *α*‐PD‐L1 was selected for antitumor immunotherapy. Because solid tumors present an immune cold state, ɑ‐PD‐L1 alone was injected into mice with only 11% and 22% inhibition of the primary tumor and distal tumors (Figure 6b,c). The AuPtAg‐GOx + 1064 nm group could inhibit the primary tumor with high efficiency (74%), but the distal tumor could only inhibit 60%. However, the AuPtAg‐GOx + 1064 nm group combined with ɑ‐PD‐L1 could inhibit the growth of both primary and distal tumors with more than 90% inhibition. After 14 d of treatment, primary tumor and distal tumor photographs are shown in Figure 6e,f, respectively. Because of the synergistic effect, the mice of AuPtAg‐GOx + 1064 nm + ɑ‐PD‐L1 group all survived within 42 d (Figure 6d) and there were almost no metastatic nodules in AuPtAg‐GOx + 1064 nm + *α*‐PD‐L1 group compared with the control group, indicating that the synergistic therapy could effectively inhibit cancer metastasis (Figure S32, Supporting Information). In addition, hematoxylin and eosin (H&E) assay showed that the tumor tissue was observably destroyed in the AuPtAg‐GOx + 1064 nm + ɑ‐PD‐L1 group (Figure 6g) and the major organs (heart, liver, spleen, lung, and kidney) of the mice showed no lesions in any group (Figure S33, Supporting Information). Moreover, there was no evident abnormality in the weight of mice during the 14d monitoring process (Figure S34, Supporting Information). Furthermore, blood biochemical experiments demonstrated that the detection parameters were in the normal range for both AuPtAg‐GOx treated and healthy mice groups (Table S2, Supporting Information), suggesting the extraordinary biosafety of AuPtAg‐GOx when used in vivo.

## 3 Conclusion

All in all, we designed an AuPtAg‐GOx nanozyme with mild photothermal properties and the ability to diminish the heat resistance of tumor cells to potentiate the anti‐tumor immune response. Upon 1064 nm laser irradiation, the tumor part would generate mild heat resulting from the AuPtAg‐GOx. Prior to this, AuPtAg‐GOx entering into tumor cells would firstly realize starvation therapy by consuming intratumoral glucose, which would suppress the content of ATP and produce H_2_O_2_. Then, the decrease in ATP concentration could hinder HSP production to render tumors less thermoresistance. Importantly, the coalescence of mild PTT with starvation therapy significantly enhances the efficacy of mild PTT with more excellent recruitment of TILs and making tumors “hot”, which could respond to immune checkpoint blockers. In vivo experiments also showed that ɑ‐PD‐L1 combined with AuPtAg‐GOx mediated mild PTT, starvation therapy and immunotherapy could better inhibit primary and distal tumors. In summary, starvation therapy‐augmented mild PTT can effectively reverse the immunosuppressive TME, indicating this strategy holds promise as a powerful tool for synergistic cancer immunotherapy.

## Conflict of Interest

The authors declare no conflict of interest.

## Supporting information

Supporting Information

## Data Availability

Research data are not shared.
