# Supplementary material for: A Noble AuPtAg‐GOx Nanozyme for Synergistic Tumor Immunotherapy Induced by Starvation Therapy‐Augmented Mild Photothermal Therapy
Source: Adv Sci (Weinh). 2022 Sep 25;9(31):2202332. doi: 10.1002/advs.202202332 (PMC9631081; doi:10.1002/advs.202202332)
Supplement: Supplementary file 1 — Supporting Information [file ADVS-9-2202332-s001.pdf]

ADVANCED SCIENCE
Open Access

Supporting Information

for *Adv. Sci.*, DOI 10.1002/advs.202202332

A Noble AuPtAg-GOx Nanozyme for Synergistic Tumor Immunotherapy Induced by Starvation Therapy-Augmented Mild Photothermal Therapy

Man Wang, Mengyu Chang, Pan Zheng, Qianqian Sun, Guangqiang Wang, Jun Lin and Chunxia Li**

Supporting Information

A Noble AuPtAg-GOx Nanozyme for Synergistic Tumor Immunotherapy Induced by Starvation Therapy-Augmented Mild Photothermal Therapy

Man Wang, Mengyu Chang, Pan Zheng, Qianqian Sun, Guangqiang Wang, Jun Lin and Chunxia Li**

EXPERIMENTAL SECTION

Materials. *L*-proline, chloroauric acid ($HAuCl_4$), chloroplatinic acid (H_2PtCl_6), silver nitrate ($AgNO_3$) and ascorbic acid (AA) were purchased from Shanghai Macklin Biochemical Co., Ltd. SH-PEG_{3000}-NH_2 was purchased from PegBio Co., Ltd (Jilin, China). GOx were purchased from Aladdin Reagent Co., Ltd (Shanghai). APC anti-mouse F4/80 (cat.123115), FITC anti-mouse F4/80 (123107), PE anti-mouse CD86 (cat. 105007), APC anti-mouse CD86 (105011), PE anti-mouse CD206 (cat. 141705), APC anti-mouse CD4 (cat. 100411), PE antimouse CD8a (cat. 100707), FITC anti-mouse CD3 (cat. 100203), FITC anti-mouse CD11c (cat. 117305), APC anti-mouse CD80 (cat. 104713), anti-CD25-APC (cat. 101909), PE antimouse FOXP3 (cat. 126403), anti-CD4-FITC (cat. 100405) and True-Nuclear™ Transcription Factor Buffer Set were purchased from BioLegend. In vivo MAb anti-mouse PD-L1 (B7-H1, cat. BE0101) were purchased from Bioxcell. All chemicals were of analytical grade and used without further purification.

Statistical Analysis. All the data are shown as the mean ± SD. Unless stated otherwise, experiments were implemented in triplicate. The significance of the difference was decided through one-way analysis of variance (*$p < 0.05$, **$p < 0.01$, ***$p < 0.001$).

Cell lines and animals. L929 (mouse fibroblast cell line), 4T1 cells (mouse breast cancer cell line) and RAW 264.7 cells were selected for cell experiments. 4T1 cells were seeded in RPMI 1640 medium supplemented with 10% FBS, penicillin (100 units mL^{-1}) and streptomycin (100 μg mL^{-1}) in 5% CO_2 at 37℃ 4T1 cells were obtained from Shanghai Institute of Biochemistry and Cell Biology, Chinese Academy of Sciences (Shanghai, P. R. China). L929 and RAW 264.7 cells purchased from Procell were seeded in DMEM medium supplemented with 10% FBS, penicillin (100 units mL^{-1}) and streptomycin (100 μg mL^{-1}) in 5% CO_2 at 37℃ Female Balb/c mice (Six weeks old) were acquired from the Center for Experimental Animals, Jilin University (Changchun, China). The procedures for the animal experiments were implemented under protocols of the National Regulation of China for Care and Use of Laboratory Animals.

Synthesis of AuPtAg nanozymes. First, 0.0576 g of *L*-proline was dissolved in 10 mL of water. Subsequently, $HAuCl_4$ (0.02 mmol), H_2PtCl_6 (0.02 mmol), $AgNO_3$ (0.02 mmol), and ascorbic acid (0.1 mmol) were sequentially added to the above solution. AuPtAg was obtained after 25 min reaction and collected by centrifugation.

Synthesis of AuPtAg-GOx. SH-PEG-NH_2 (10 mg) was dispersed in 15 mL of water, 10 mL of AuPtAg (1 mg mL^{-1}) was injected into the above solution to obtain AuPtAg-PEG-NH_2 after 24 h stirring. After that, GOx (1 mg), EDC (10 mg) and NHS (10 mg) were dissolved in 10 mL of water. After activation for 45 min, AuPtAg-PEG-NH_2 solution was added into the above solution to react for 1 h. AuPtAg-GOx was collected by centrifugation.

Photothermal properties of AuPtAg-GOx NPs. To measure the photothermal effect of AuPtAg-GOx, AuPtAg-GOx with different concentration (0, 125, 250, 500, and 1000 μg mL^{-1}) was exposed to NIR light for 300 s (808 nm or 1064 nm, 0.5 W cm^{-2}). The real-time temperature was recorded every 30 s by infrared thermal camera. Then, the photothermal effect of AuPtAg-GOx (1000 μg mL^{-1}) was studied by irradiation with different power (0.25, 0.5 and 1 W cm^{-2}).

Calculation of Photothermal Conversion Efficiency (η): The photothermal conversion efficiency is calculated by formula (1):

$$\eta = \frac{hS(T_{max,NP} - T_{surr}) - Q_{dis}}{I(1 - 10^{-A_{808}})} \qquad (1)$$

where h is the heat transfer coefficient, S is the surface area of the container, T_{max} is the maximum temperature of the solution, T_{surr} is the surrounding temperature, I is the laser power density, A_{808} and A_{1064} are the absorption value of the material at 808 nm and 1064 nm, respectively. Q_{dis} is the heat generated after water and container absorbs light. To calculate hS, equation (2) (3) was introduced:

$$Q_{dis} = hS(T_{max,H_2O} - T_{surr}) \qquad (2)$$

$$\tau_s = \frac{m_D C_D}{hS} \qquad (3)$$

m_D is the mass of water, C_D is the heat capacity of water (4.2 $J \cdot g^{-1} \cdot {}^{\circ}C^{-1}$), τ_s is the sample system time constant, which was calculated by formula (4) (5):

$$t = -\tau_s ln\theta \qquad (4)$$

$$\theta = \frac{T_{surr} - T}{T_{surr} - T_{max}} \qquad (5)$$

CAT-like activity of AuPtAg-GOx. AuPtAg-GOx (100 μg mL^{-1}) and H_2O_2 (3 mmol L^{-1}) were mixed to 3 mL of PBS (pH 6.75). The O_2 content was detected by a dissolved oxygen meter (JPB-607A).

GOx activity of AuPtAg-GOx. AuPtAg-GOx (250 μg mL^{-1}) and glucose (0.5 mg mL^{-1}) were mixed to 5 mL of PBS (pH 6.75). Supernatants were taken at 0, 20, 40, and 60 min of reaction to test glucose concentrations via the Glucose Assay Kit with O-toluidine (Beyotime, S0201S).

Cellular uptake. 4T1 cells were seeded in 12-well plate at a density of 8×10^4 cells per well for 12 h. The AuPtAg-GOx-RhB (50 μg mL^{-1}) was added to the cells according to the timing of the countdown. After incubation for 6 h, the cellular uptake was observed by a fluorescence microscope. Then, 4T1 cells incubated with AuPtAg-GOx-RhB for various times (0 h, 0.5 h, and 6 h) were digested and the fluorescence intensity of AuPtAg-GOx-RhB was tested by flow cytometry.

Cell compatibility. In total, 8000 L929 or 4T1 cells were seeded into 96 well plates and incubated with different concentrations (0, 100, 200, 300, 400 and 500 μg mL^{-1}) of AuPtAg-PEG dispersed in DMEM or RPMI for 24 h. Relative cell viabilities were detected by the standard 3-(4,5-dimethylthiazol-2-yl)-2,5-diphenyltetrazolium bromide (MTT) assay.

In vitro cytotoxicity study of AuPtAg-GOx. The 4T1 cells (8000 cells per well) were seeded into a 96-well plate and incubated with different concentrations (0, 50, 100 and 200 μg mL^{-1}) of AuPtAg-GOx for 24 h. After 24 h of incubation, the excess unbound materials were washed for three times with PBS, then 10 μL of 3-(4, 5-dimethylthiazol-2-yl)-2, 5-diphenyltetrazolium bromide (MTT) solution was added into each well. After 4 h of treatment, the solution was discarded, and 150 μL of dimethyl sulfoxide (DMSO) was added to dissolve crystals. Finally, the absorbance was measured at 490 nm using a microplate reader.

Intracellular cytotoxicity study. 4T1 cells (8000 cells per well) were seeded in a 96-well plate for 24 h. Subsequently, AuPtAg-PEG and AuPtAg-GOx (200 μg mL^{-1}) was added. After 4 h of incubation, the 4T1 cells were irradiated for 5 min with 1064 nm laser (0.5 W cm^{-2}). Finally, the cells were incubated for 20 h and the cell viability was studied by MTT assay.

Live-dead cell staining experiments: 4T1 cells were seeded in 12-well plate at a density of 1×10^5 cells per well for 12 h. AuPtAg-PEG and AuPtAg-GOx (200 μg mL^{-1}) was added. After 4 hours of incubation, the 4T1 cells were irradiated for 5 min with 1064 nm laser (0.5 W cm^{-2}). Then, calcein AM and pyridine iodide (PI) were added to dye cells. Inverted fluorescence microscope system was used to detect fluorescence.

Western blot Analysis. 4T1 cells were seeded into culture plates at a density of 3×10^6 cells per dish and treated with A: control, B: 42°C incubation; C: AuPtAg-PEG (100 μg mL^{-1}) + 1064 nm; D: AuPtAg-GOx (100 μg mL^{-1}) + 1064 nm for 12 h, respectively. Subsequently, the cells were collected for a standard western blot process.

In vitro Macrophage Polarization. RAW 264.7 macrophages were cultured with IL-4 (25 ng mL^{-1}) for 12 h to induce M2 polarization. The residues of 4T1 cells after different treatments with untreated, AuPtAg-GOx (200 μg mL^{-1}), AuPtAg-PEG + 1064 nm and AuPtAg-GOx + 1064 nm (0.5 W cm^{-2}) were used to incubate with M2 macrophages for another 12 h using a transwell system. Afterwards, RAW 264.7 macrophages were collected and stained by APC anti-CD86 and PE anti-CD206 antibodies, and then was measured by flow cytometry (Guava EasyCyte). The level of cytokines (IL-12 and IL-10) in supernatants were collected and detected through ELISA assay.

In vivo Antitumor Immunity. Mice were received subcutaneous injection of 4T1 cells at the right (5×10^6 cells). When tumor grew to about 50 mm^3, Balb/c mice were classified into four groups at random (n = 5): (1) control, (2) AuPtAg-GOx, (3) AuPtAg-PEG + 1064 nm, (4) AuPtAg-GOx + 1064 nm. Mice were injected intravenously with nanocomposites (100 µL, 20 mg kg^{-1}). For macrophage polarization analysis, the tumors of mice were collected, homogenized in PBS, and filtered to gain single-cell suspension. Then, the cell suspension of tumors was stained with APC anti-F4/80, PE anti-CD86 and PE antiCD206 antibodies for macrophage phenotype analysis by flow cytometry.

For dendritic cells maturation analysis, the lymph nodes of mice were collected, homogenized in PBS, and filtered to gain single-cell suspension. Afterwards, the cell suspension of lymph nodes was stained with FITC anti-CD11c, PE anti-CD86 and APC anti-CD80 antibodies for dendritic cells maturation analysis by flow cytometry.

For T cells activation analysis, the spleens were homogenized in PBS to obtain single-cell suspension, and washed with erythrocyte lysis buffer to remove erythrocytes. Then, the cell suspensions of spleens and tumor were stained by FITC anti-CD3, PE anti-CD8a and APC anti-CD4, and analyzed by flow cytometry.

To analyze Tregs, the spleens were homogenized in True-Nuclear™ Transcription Factor Buffer Set to obtain single-cell suspension, the suspension was stained by FITC anti-mouse CD4, anti-CD25-APC and PE anti-mouse FoxP3, and analyzed by flow cytometry. The content of cytokines (TNF-α and IL-6) in the supernatant was detected using ELISA kits.

In vivo anti-tumor effect in a bilateral 4T1 tumor model. A bilateral 4T1 tumor model was constructed for researching in vivo anti-tumor effects of the AuPtAg-GOx. For primary tumors, 4T1 cells were inoculated into Balb/c mice (1×10^6 cells per mouse) at the right back. After three days, 4T1 cells were inoculated into Balb/c mice (5×10^5 cells per mouse) at the left back as distant tumors. And after four days, the 4T1 tumor-bearing Balb/c mice were randomly allocated into 5 groups (n = 5): (1) control, (2) α-PD-L1, (3) AuPtAg-GOx, (4) AuPtAg-PEG + 1064 nm, (5) AuPtAg-GOx + 1064 nm (100 μL, 20 mg kg^{-1}) was intravenously injected into mice when the tumor volume grew to 50 mm^3 (day 0). Then, mice were intravenously injected with 20 μg of α-PD-L1 in 0.1 mL of PBS on day 1, 3, 5, and 7. The tumor sizes were monitored every 2 days and bodyweights were recorded at the same time. Tumor volume = $length \times width^2/2$.

Anti-metastatic studies in a 4T1 tumor model. In order to evaluate the anti-metastasis ability of the system, a lung metastasis model was established. Briefly, 4T1 tumor bearing Balb/c mice were randomly divided into five groups (n = 5) and injected with 1) saline, 2) AuPtAg-GOx, 3) AuPtAg-PEG + 1064 nm, 4) AuPtAg-GOx + 1064 nm and 5) AuPtAg-GOx + 1064 nm + α-PD-L1. The α-PD-L1 was injected into mice by tail vein on days 1, 3, 5, and 7 of treatment. On day 8, 100 μL of PBS containing 4×10^5 4T1 cells were injected into mice by tail vein for lung metastasis study. On day 23, all the mice were sacrificed, and the lung were surgical removed and collected for H&E staining.

H&E staining methods. For histology assay, major organs (heart, liver, spleen, lung and kidney) and tumors were harvested from mice. The collected tumors and organs were fixed in 10% paraformaldehyde, embedded in paraffin, sectioned into ~4 μm, and stained with H&E. In detail, paraffin sections were dewaxed first in xylene for 5 min, replaced with fresh xylene for another 5 min, after that the sections were treated with absolute ethanol for 5 min, 90% (*V*/*V*) ethanol for 2 min, 80% (*V*/*V*) ethanol for 2 min, 70% (*V*/*V*) ethanol for 2 min, distilled water for 2 min. Then, stain the glass slides of tissue in hematoxylin solution for 5 min, rinse it in running tap water for 10 min and purified water for another 30 s. Whereafter, stain the glass slides of tissue in eosin solution for 5 min. Immerse the glass slides of tissue in 70% (*V*/*V*) ethanol for 10 s, 80% (*V*/*V*) ethanol for 10 s, 90% (*V*/*V*) ethanol for 10 s, 100% (*V*/*V*) ethanol for 10 s, xylene for 5 min, and xylene for 5 min. Finally, seal the glass slides of tissue with neutral gum. The glassslides with H&E staining are measured by a microscope.

	Raw material ratio (mol)			Product ratio (mol)			Pt (%)
	Au	Pt	Ag	Au	Pt	Ag	
1	1	1	1	1	1	1.22	31
2	1	1	0.5	1	0.9	0.58	36.3
3	1	1	2	1	1.13	2.27	25.7
4	1	0.5	1	1	0.6	1.51	19.3
5	1	2	1	1	1.63	1	44.9
6	0.5	1	1	1	2.33	8.53	19.6
7	1	0.5	0.5	1	0.45	0.65	21.4

Table S1. The raw material ratio (mol), product ratio (mol) and Pt percentage detected by ICP in different Au/Pt/Ag ratios.

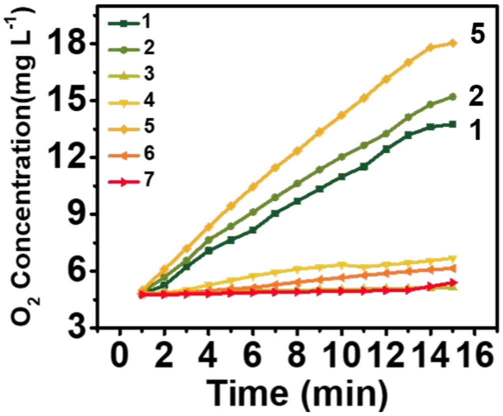

Figure S1. Comparison of catalase-like activity of different molar ratios of AuPtAg.

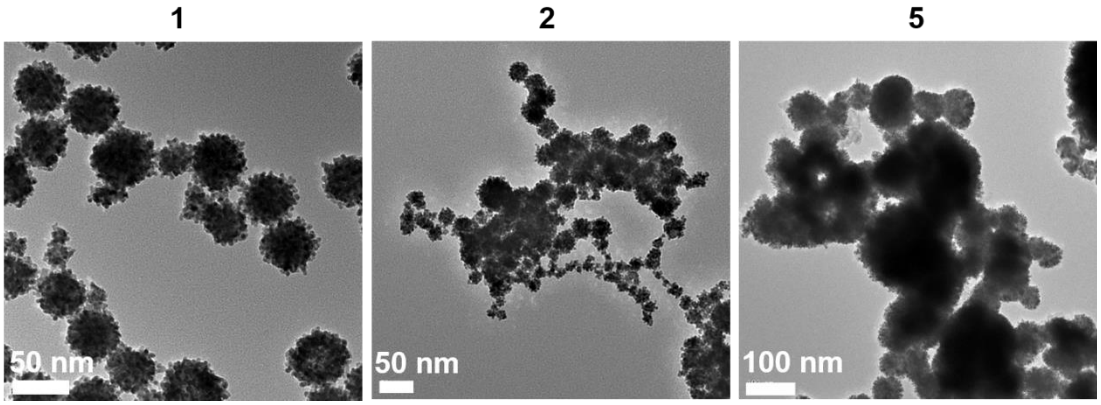

Figure S2. TEM of materials 1, 2 and 5 with the molar ratios of Au, Pt and Ag of 1: 1: 1.22, 1: 0.9: 0.58 and 1: 1.63: 1, respectively.

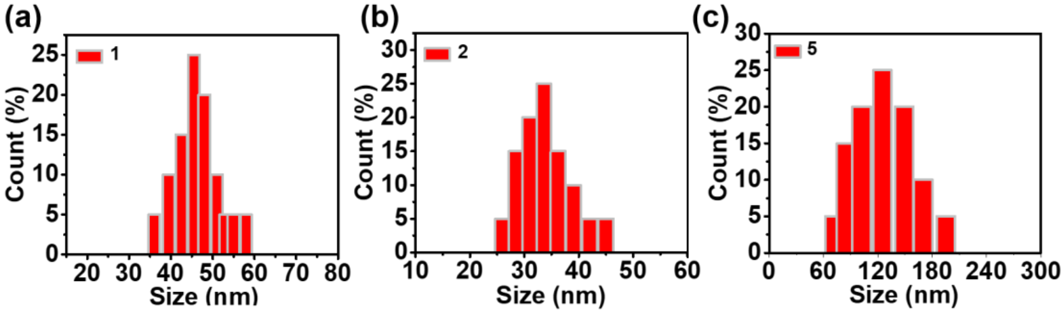

Figure S3. Size distribution of materials 1, 2 and 5 with the molar ratios of Au, Pt and Ag of 1: 1: 1.22, 1: 0.9: 0.58 and 1: 1.63: 1, respectively.

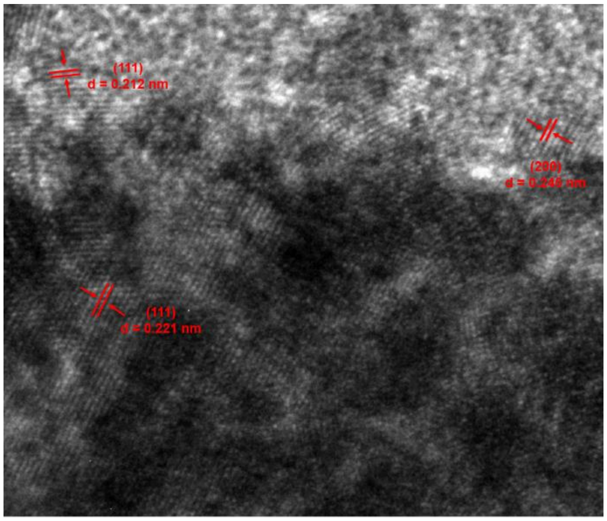

Figure S4. HRTEM image of AuPtAg nanozymes.

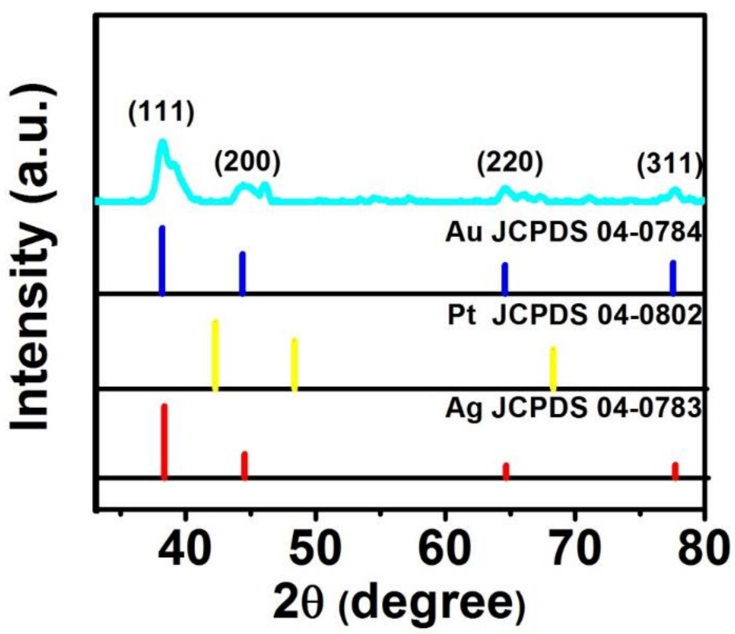

Figure S5. XRD spectra of AuPtAg nanozymes.

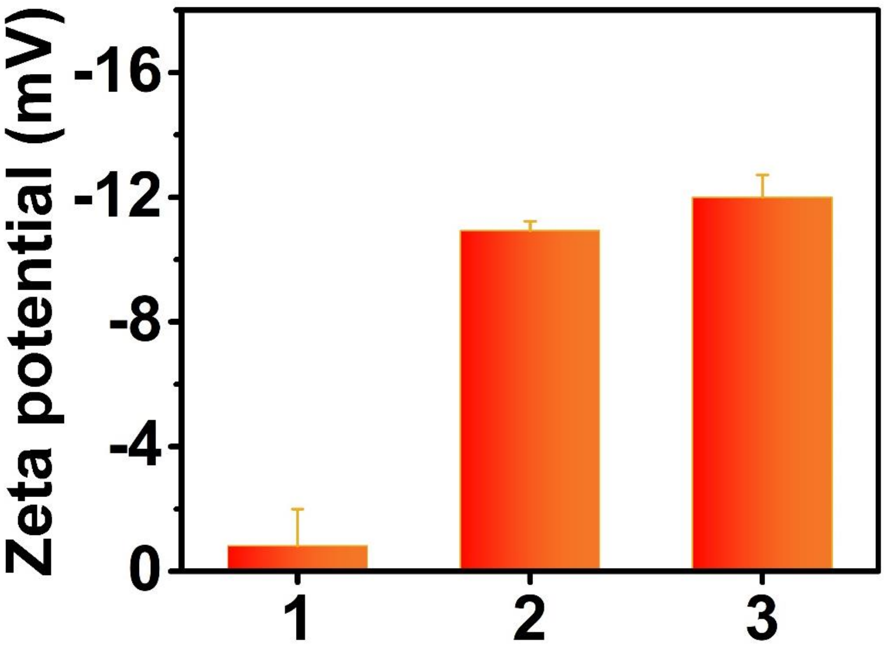

Figure S6. The zeta potential of AuPtAg (1), AuPtAg-PEG-NH_2 (2) and AuPtAg-GOx (3).

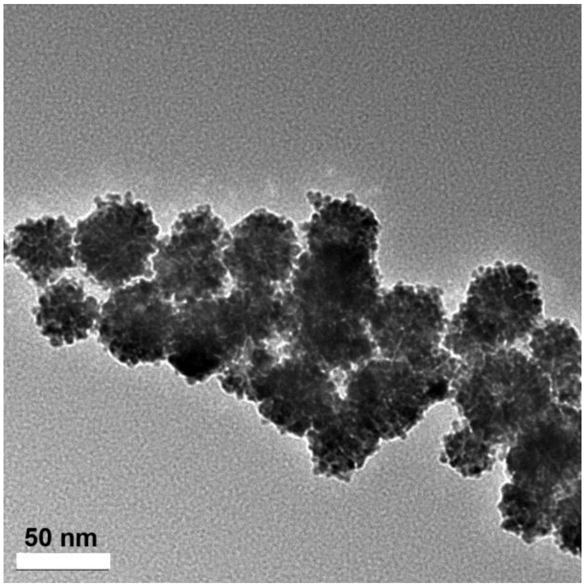

Figure S7. The TEM image of AuPtAg-GOx.

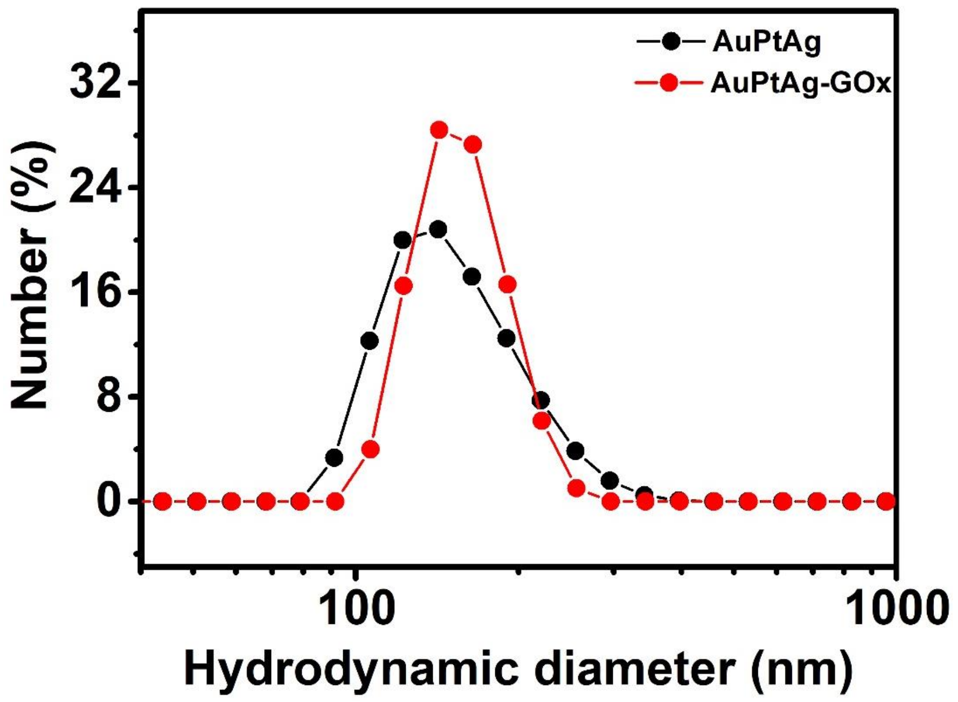

Figure S8. Hydrodynamic diameter of AuPtAg and AuPtAg-GOx tested in water, respectively.

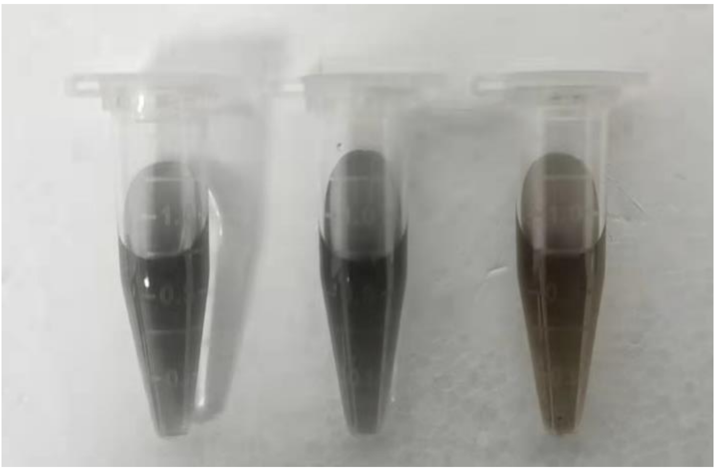

Figure S9. Photographs of AuPtAg-GOx NPs in water (1), PBS (2) and RPMI containing 10% fetal bovine serum (FBS).

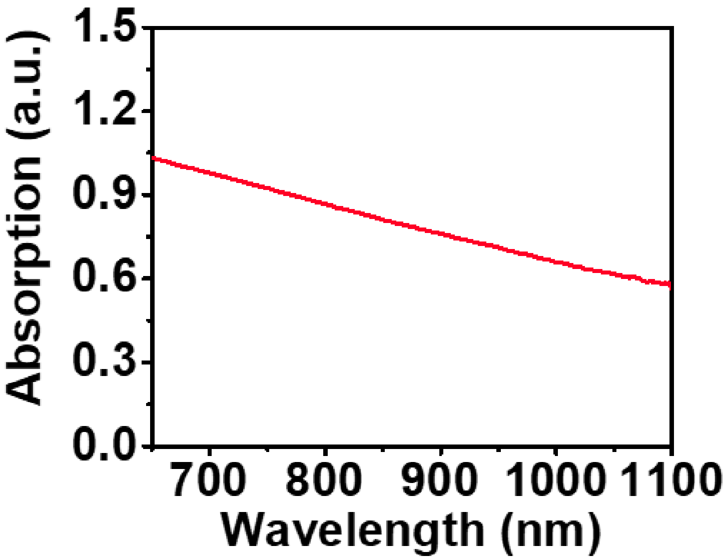

Figure S10. UV–Vis–NIR absorption spectra of AuPtAg.

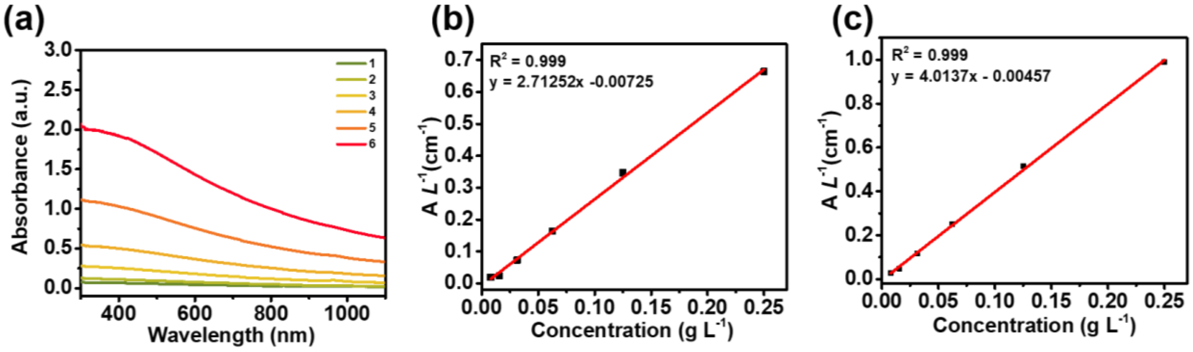

Figure S11. (a) UV–vis–NIR absorbance spectra of the AuPtAg-GOx NPs at varied concentrations. Mass extinction coefficient of AuPtAg-GOx NPs at (b) 1064 nm and (c) 808 nm.

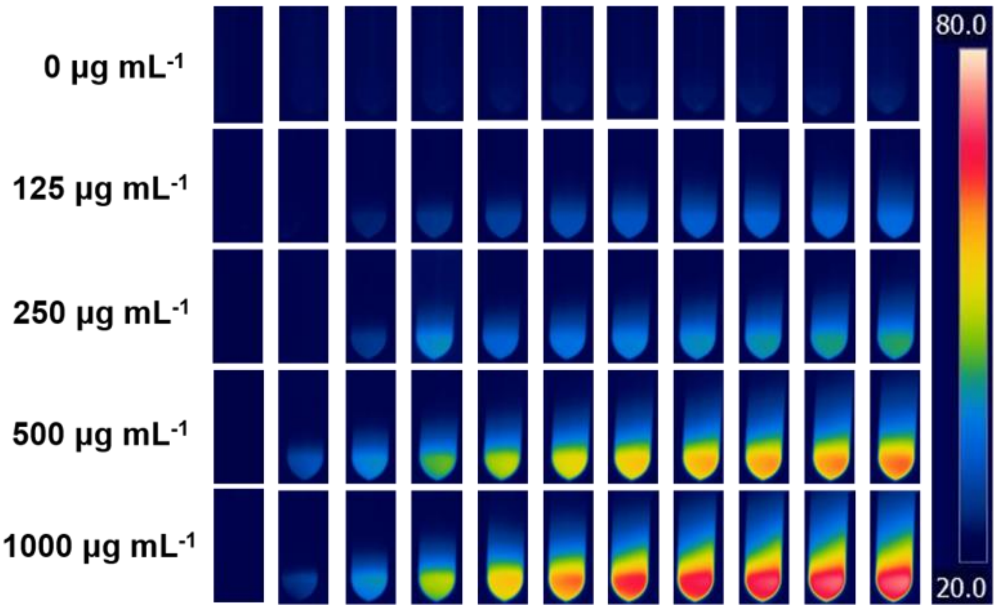

Figure S12. Thermal images of AuPtAg-GOx with different concentrations exposed to 808 nm laser (0.5 W cm^{-2}) irradiation for 5 min.

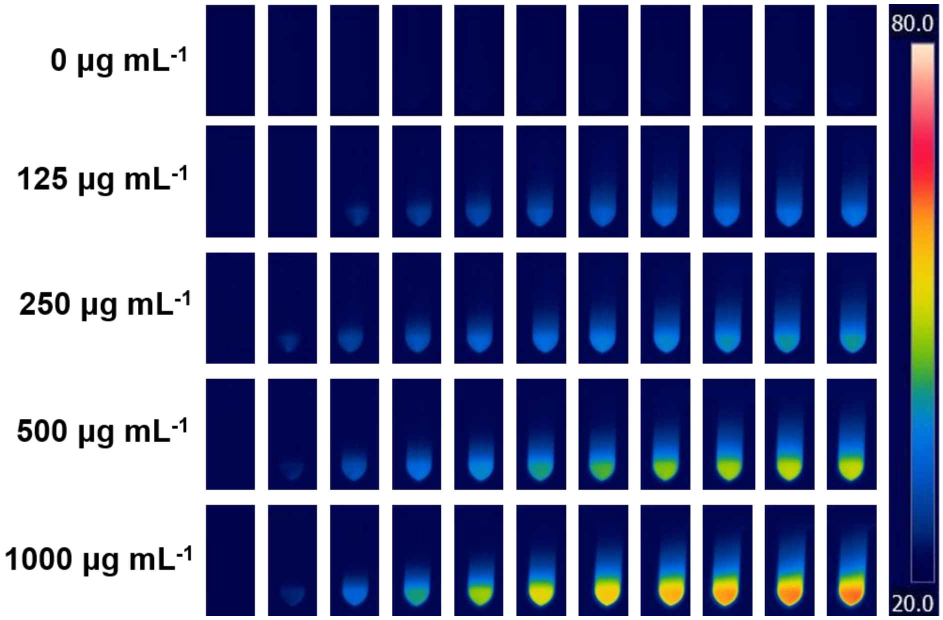

Figure S13. Thermal images of AuPtAg-GOx with different concentrations exposed to 1064 nm laser (0.5 W cm^{-2}) irradiation for 5 min.

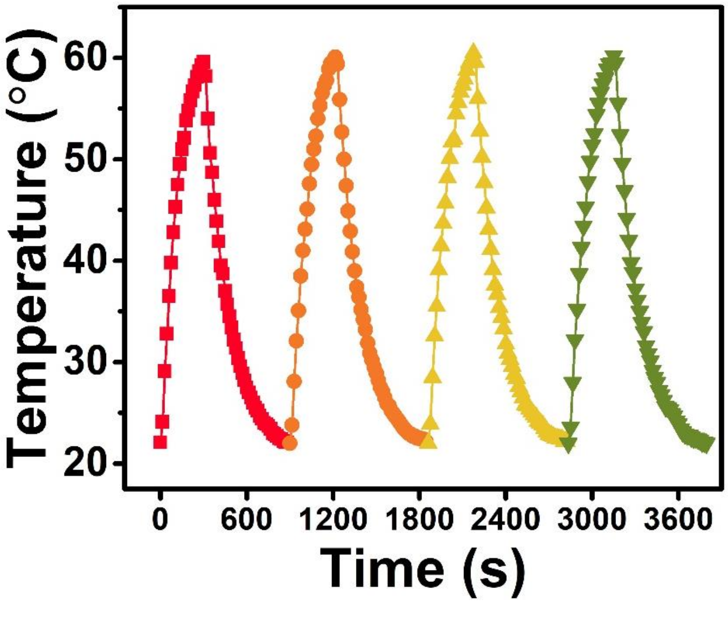

Figure S14. Temperature changes of AuPtAg-GOx NPs during four on/off cycles of laser irradiation (1064 nm, 0.5 W cm^{-2}).

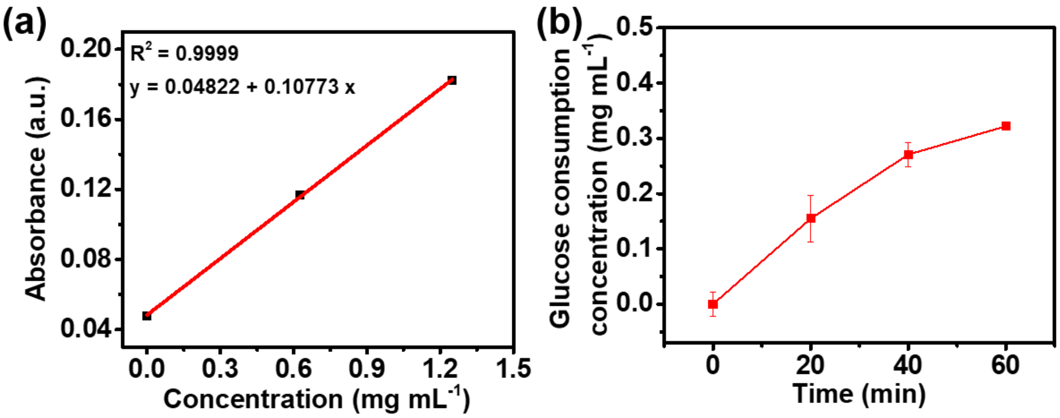

Figure S15. (a) The standard curve of glucose absorption intensity as a function of concentration. The absorption intensity was measured at 630 nm. (b) Growth curve of glucose consumption concentration with reaction time.

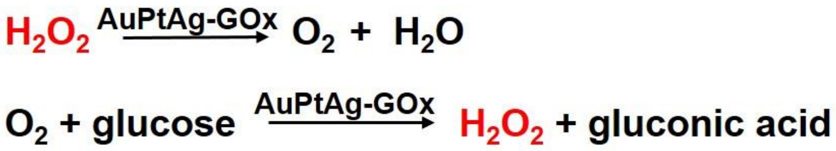

Figure S16. The cascade reaction equation of AuPtAg-GOx.

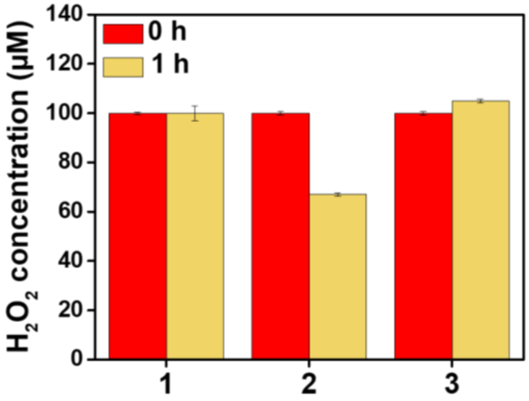

Figure S17. The H_2O_2 concentration at the beginning of the reaction and after 1 h among different groups. 1) AuPtAg + glucose; 2) AuPtAg + glucose + H_2O_2; 3) AuPtAg-GOx (50 μg mL^{-1}) + glucose (4 mM) +

H_2O_2 (100 μM). The H_2O_2 content was measured in AuPtAg-GOx plus glucose solution by Hydrogen Peroxide Assay Kit (Beyotime, S0038). As shown in Figure S17, The H_2O_2 content of AuPtAg + glucose + H_2O_2 group decreased by 33% but increased by 5% in the AuPtAg-GOx + glucose + H_2O_2 group. The above proves that AuPtAg can consume H_2O_2, while the AuPtAg-GOx system does not consume ROS because GOx can produce H_2O_2.

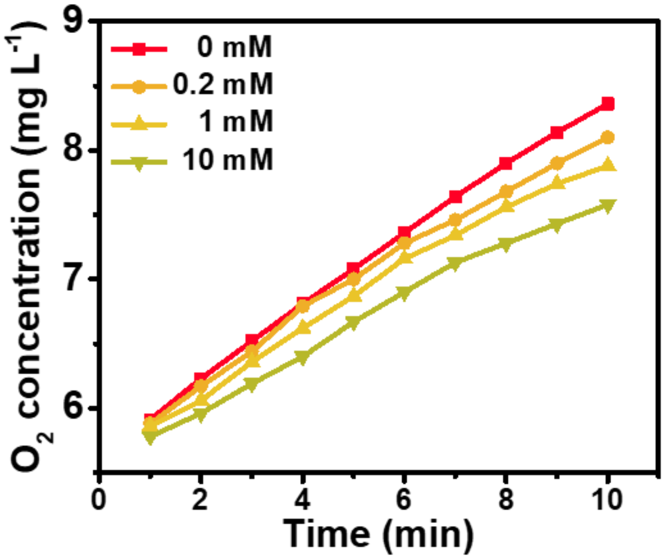

Figure S18. Oxygen generation curves of AuPtAg (50 μg mL^{-1}) in H_2O_2 (3 mM) solution under different glucose concentrations.

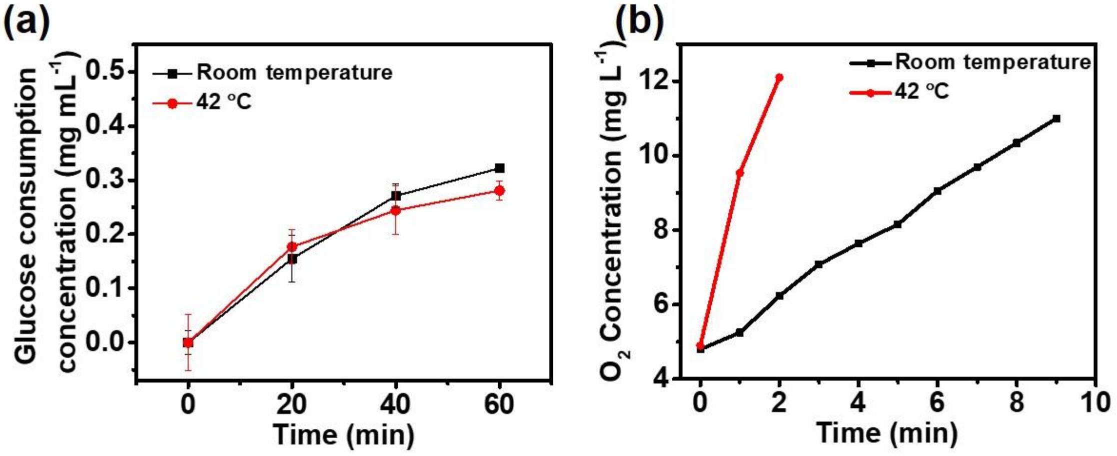

Figure S19. (a) Comparison of glucose consumption capacity of AuPtAg-GOx at different temperatures. (b) The amount of oxygen production of AuPtAg in different temperatures.

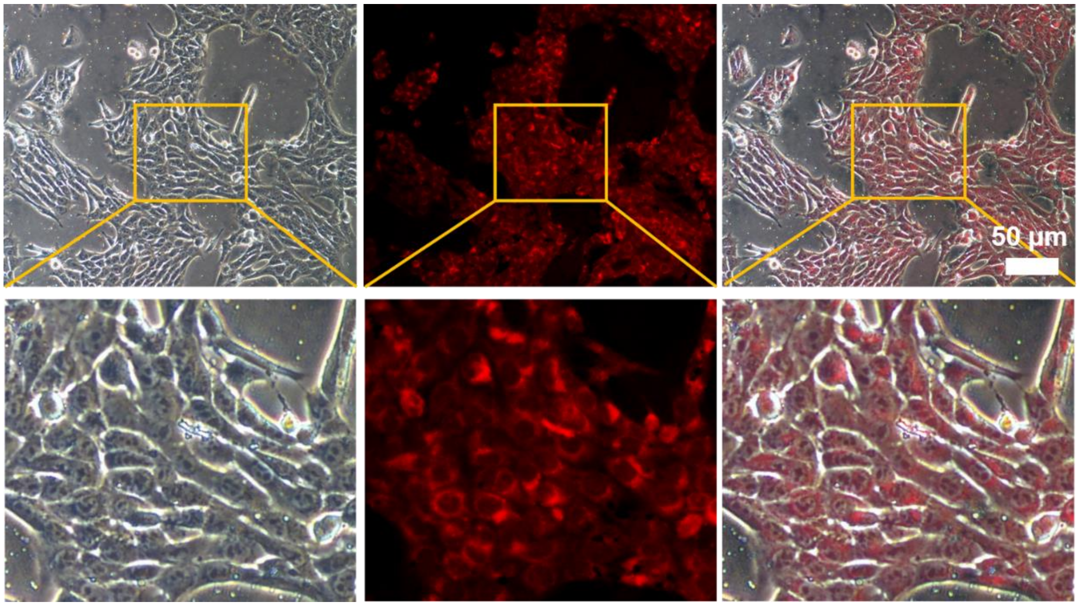

Figure S20. The microscopy images of 4T1 cells after incubation with 50 μg mL^{-1} of AuPtAg-GOx-Rhodamine B for 6 h.

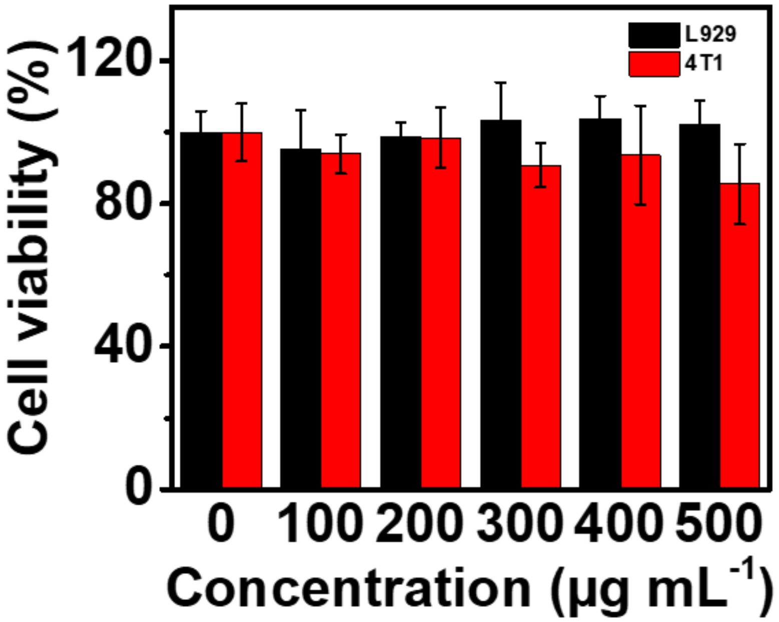

Figure S21. L929 and 4T1 cells viability incubated with AuPtAg-PEG for 24 h at different concentrations.

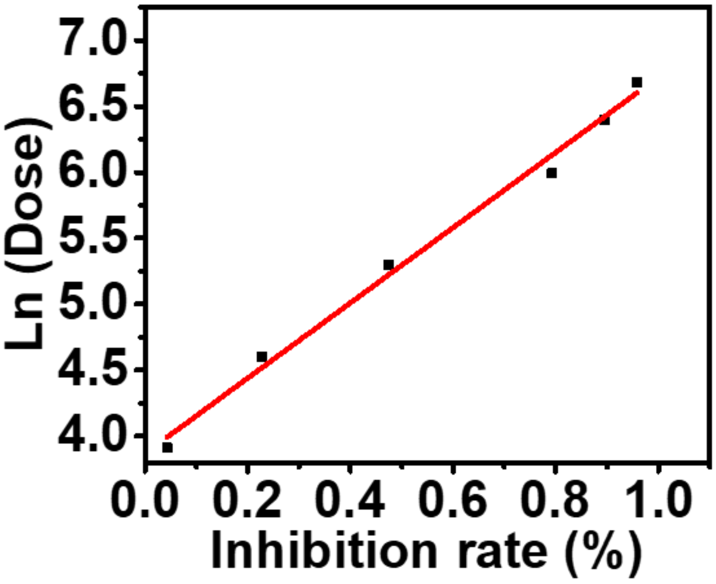

Figure S22. Plot of the ln of the concentration of AuPtAg-GOx versus inhibition rate.

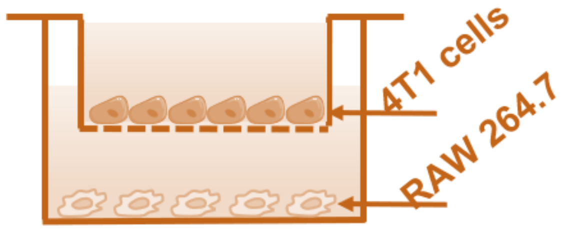

Figure S23. (a) A scheme illustrating the transwell insert system.

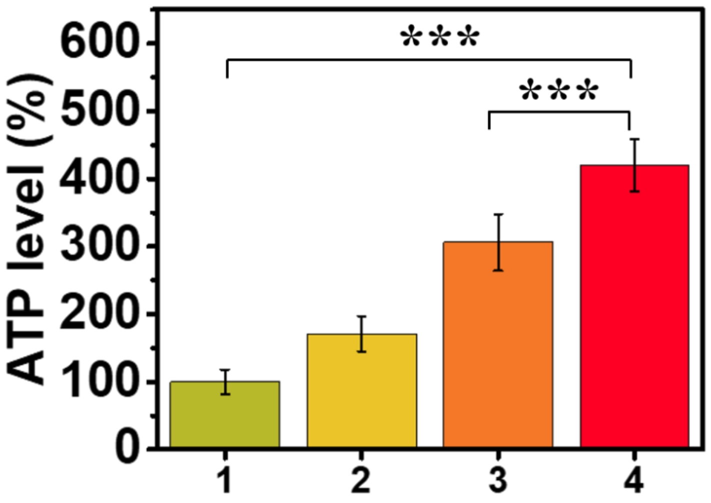

Figure S24. The release of ATP from tumor cells after different treatments: 1) control, 2) AuPtAg-GOx, 3) AuPtAg-PEG + 1064 nm (0.5 W cm^{-2}), 4) AuPtAg-GOx + 1064 nm (0.5 W cm^{-2}). Data were presented as mean ± s.d. (n = 5), ****p* < 0.001 (two tailed *t*-test).

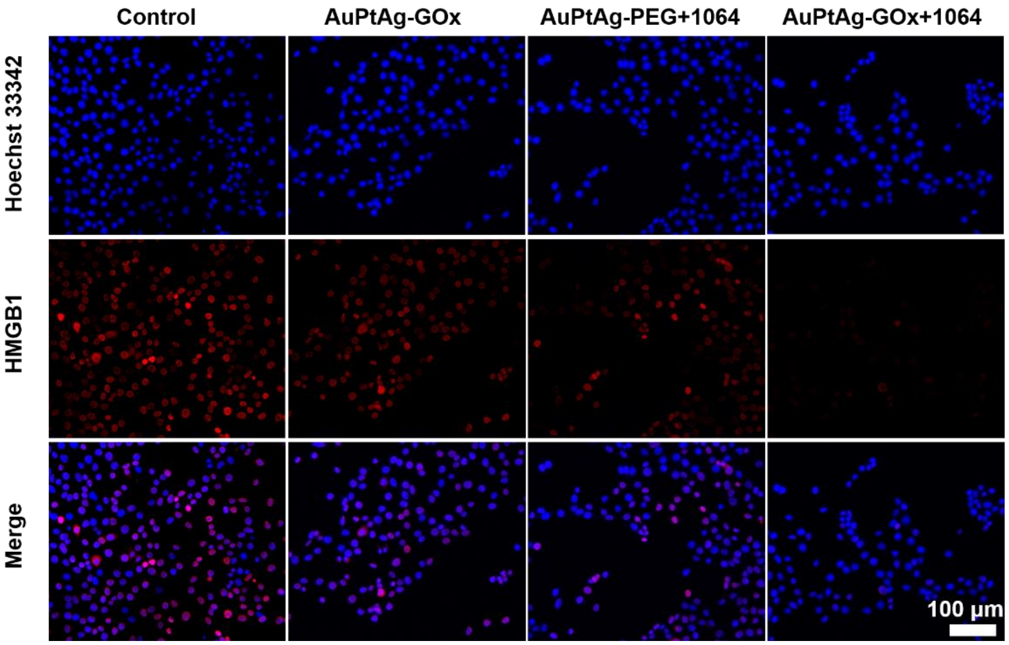

Figure S25. Fluorescence microscopy images of HMGB1 release after different treatments.

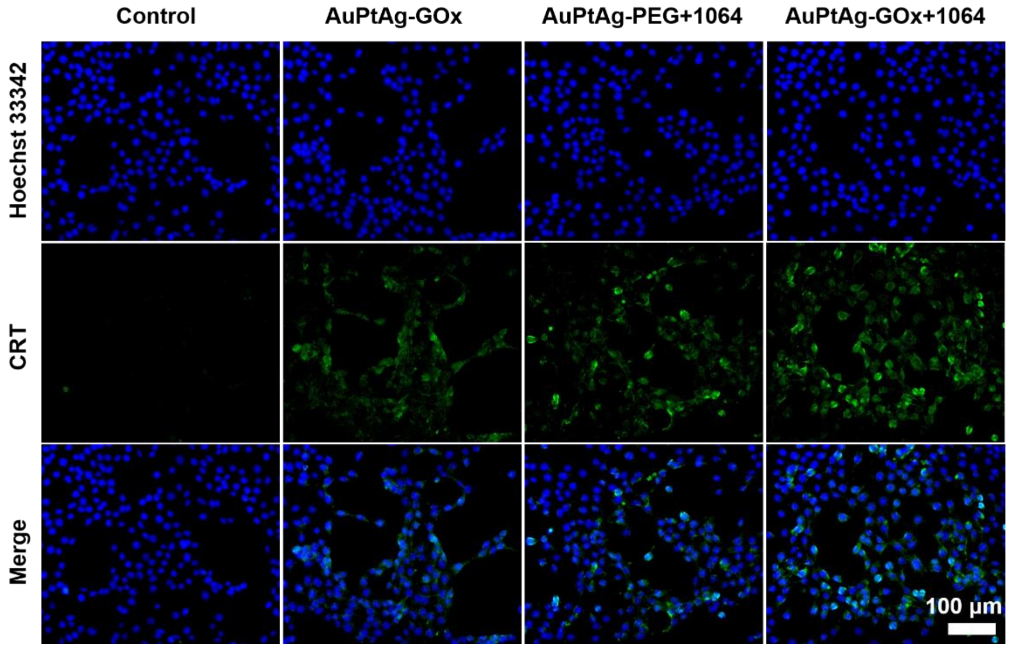

Figure S26. Fluorescence microscopy images of CRT exposure on the surface of the membrane after different treatments.

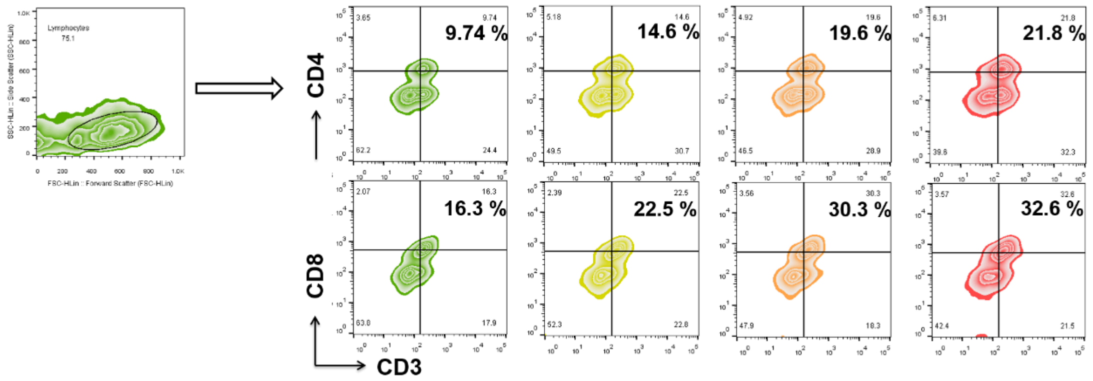

Figure S27. Gating strategy of $CD3^+CD4^+CD8^+$ T cells collected from spleens for flow cytometry analysis.

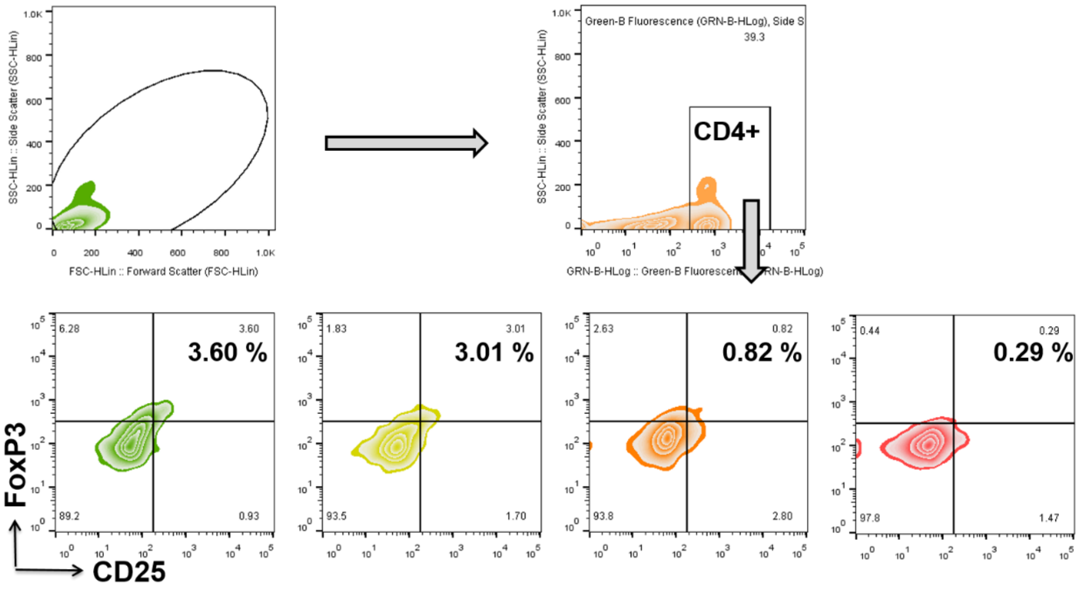

Figure S28. Gating strategy of Tregs collected from spleens for flow cytometry analysis.

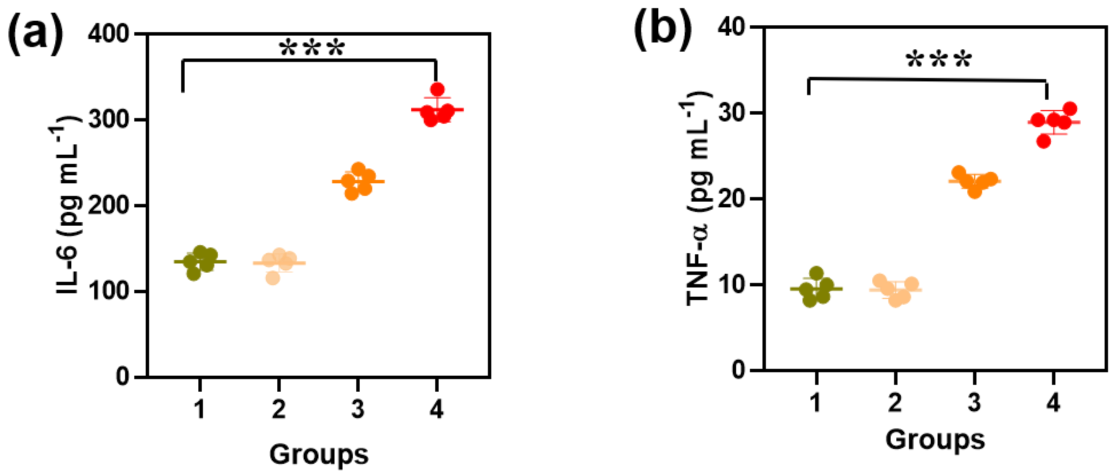

Figure S29. The level of (a) IL-6 and (b) TNF-α secreted by splenocytes obtained from immunized mice. Groups: 1) control, 2) AuPtAg-GOx, 3) AuPtAg-PEG + 1064 nm (0.7 W cm^{-2}), 4) AuPtAg-GOx + 1064 nm (0.7 W cm^{-2}). Data were presented as mean ± s.d. (n = 5), ****p* < 0.001 (two-tailed *t*-test).

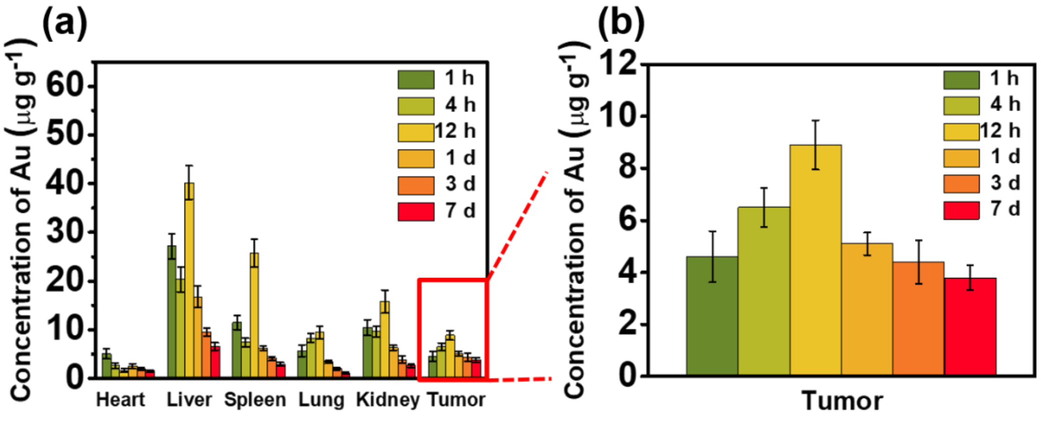

Figure S30. (a) Bio-distribution of Au in major organs and tumors of mice after injection of AuPtAg-GOx (20 mg kg^{-1}, 100 μL) at different time points. (b) The enlarged figure of Bio-distribution of Au in the tumors.

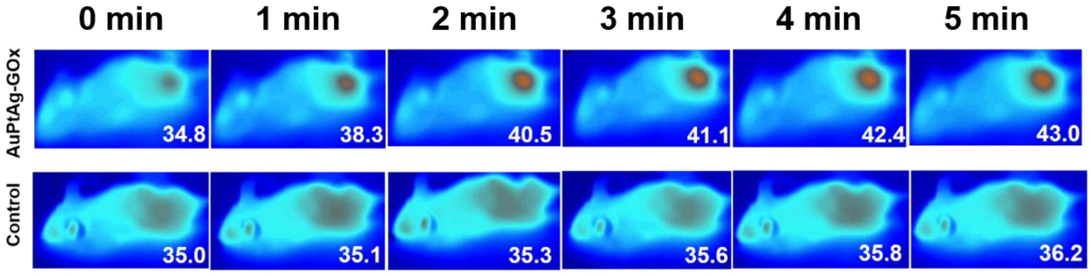

Figure S31. The thermal images of 4T1 tumor-bearing mice under 1064 nm laser irradiation (0.7 W cm^{-2}) after 12 h of intravenously injected with saline or AuPtAg-GOx (20 mg kg^{-1}, 100 μL).

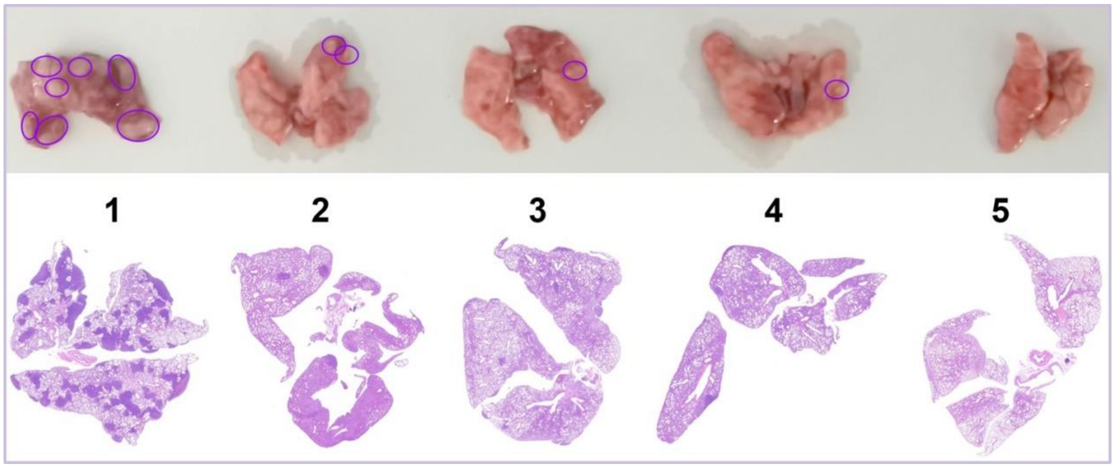

Figure S32. Representative images of lung tissues from different treatment groups, with metastatic nodules indicated by purple circles and H&E staining of lung tissues with different treatments. Groups: 1) saline, 2) AuPtAg-GOx, 3) AuPtAg-PEG + 1064 nm (0.7 W cm^{-2}), 4) AuPtAg-GOx + 1064 nm (0.7 W cm^{-2}) and 5) AuPtAg-GOx + 1064 nm + α-PD-L1 (0.7 W cm^{-2}).

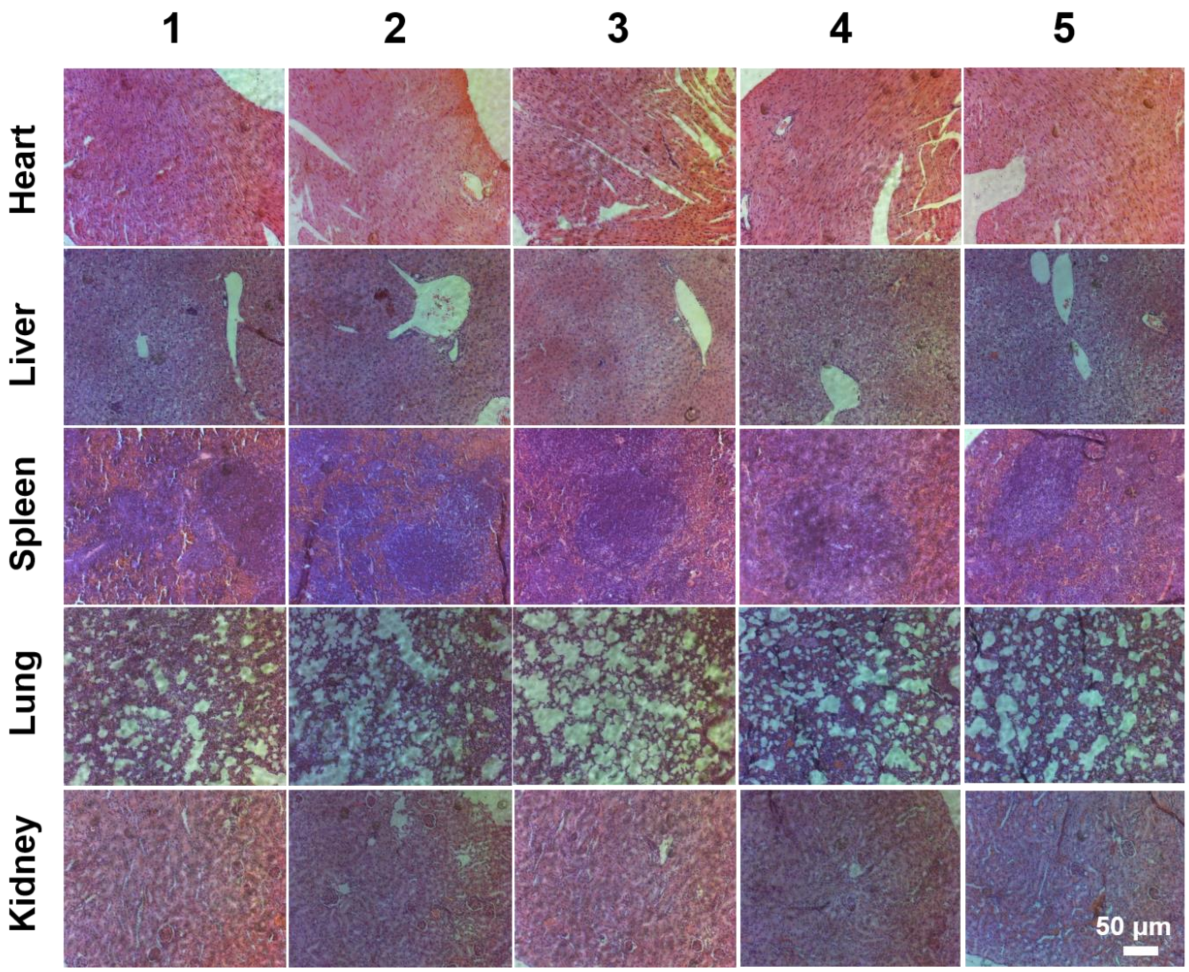

Figure S33. The H&E stained images of major organs after the different treatments. Groups: 1) control, 2) AuPtAg-GOx, 3) AuPtAg-PEG + 1064 nm (0.7 W cm^{-2}), 4) AuPtAg-GOx + 1064 nm (0.7 W cm^{-2}). 5) AuPtAg-GOx + 1064 nm + α-PD-L1 (0.7 W cm^{-2}).

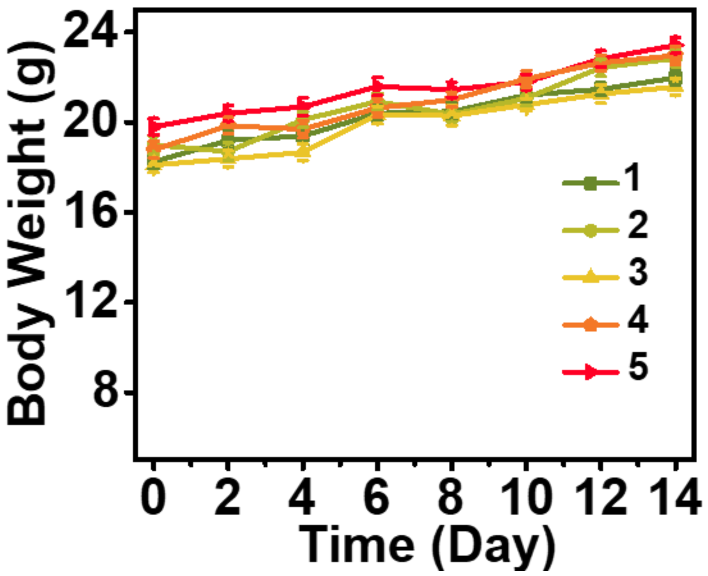

Figure S34. Change curves for the body weight of mice (days 1–14).

	Reference range	Control	14 day
ALT (U L^{-1})	27 ~ 195	69.3±3.1	90±5.3
AST (U L^{-1})	43 ~ 397	130.2±4.4	138.8±4.9
ALP (U L^{-1})	105 ~ 370	145±9.3	164±9.6
BUN (mmol L^{-1})	5 ~ 26	11.2±1.2	12.8±1.89
CREA (mmol L^{-1})	18 ~ 44	29.3±1.4	27.7±1.1
MCV (fL)	42.6 ~ 55.6	48.5±0.5	50±1.4
MCH (pg)	13.0 ~ 16.8	15.9±0.2	16.7±0.1
WBC (10^9/L)	3.90 ~ 13.94	10.2±0.6	11.7±1.1
RBC (10^{12}/L)	7.37 ~ 11.50	9.2±0.2	10.2±0.5
HCT (%)	37.2 ~ 58.0	39.6±1.2	48.2±3

Table S2. Blood biochemical and haematological analysis of the healthy mice intravenously injected with normal saline or AuPtAg-GOx (20 mg kg^{-1}, 100 μL) at 14 days post-injection.
